# Diversity of lake bacteria promotes human echovirus inactivation

**DOI:** 10.1128/aem.02366-24

**Published:** 2025-01-17

**Authors:** Andrii Romanenko, Hannes Peter, Josephine Meibom, Mark A. Borchardt, Tamar Kohn

**Affiliations:** 1 Laboratory of Environmental Virology, Environmental Engineering Institute (IIE), School of Architecture, Civil and Environmental Engineering (ENAC), Ecole Polytechnique Fédérale de Lausanne (EPFL) 111839 , Lausanne, Switzerland; 2 River Ecosystems Laboratory, Environmental Engineering Institute (IIE), School of Architecture, Civil and Environmental Engineering (ENAC), Ecole Polytechnique Fédérale de Lausanne (EPFL) 111839 , Sion, Switzerland; 3 U.S. Department of Agriculture-Agricultural Research Service, Laboratory for Infectious Disease and the Environment 17123 , Marshfield, Wisconsin, USA; The University of Queensland, Brisbane, Queensland, Australia

**Keywords:** virus inactivation, waterborne virus, lake microbial community, biodiversity ecosystem service, echovirus

## Abstract

Human enteric viruses can remain infective in surface waters for extended periods of time, posing a public health risk. Microbial activity contributes to the inactivation of waterborne enteric viruses, but while individual bacteria–virus interactions have been characterized, the importance of microbial diversity remains unknown. Here, we experimentally manipulated the diversity of bacterial communities from Lake Geneva across three seasons using a dilution-to-extinction approach and monitored the inactivation and genome decay of echovirus 11, a member of the *Enterovirus* genus. Long-read sequencing of the 16S rRNA gene revealed diversity gradients ranging between 373 and 2,722 bacterial species. Compared to sterile controls, echovirus 11 inactivation was enhanced by the presence of active bacteria and depended both on season and sample dilution. Throughout all seasons, the highest inactivation (between 3.0 and 7.9 log_10_ fold reduction in infectivity over 96 h) was observed in the least diluted incubations (i.e., the highest bacterial richness). Genome decay exhibited a 24-h lag and was less pronounced than the corresponding infectivity loss (ranging between 2.3 and 3.8 log_10_ fold over 96 h), indicating that microbial inactivation primarily targets the echovirus 11 capsid. We found a positive-saturating relationship between bacterial species richness and viral inactivation, suggesting functional redundancy and pointing toward the importance of rare species for viral inactivation. Biomarker analysis revealed several clades of bacteria, particularly members of *Chitinophagaceae*, to be significantly associated with echovirus 11 inactivation. Overall, these findings suggest that high microbial diversity enhances the capacity of surface waters to rid themselves of contamination by enteric viruses and hence protects public health.

**IMPORTANCE:**

Human enteric viruses in natural waterbodies pose a public health risk. Microorganisms, particularly bacteria, contribute to the inactivation of enteroviruses, thereby mitigating this risk. We use experimental manipulations of lake water bacterial diversity to unravel the importance of diversity for the inactivation of echovirus 11, a model human pathogen. Our findings suggest that bacterial diversity is important for echovirus 11 inactivation and that specific, but numerically rare, bacteria present in the surface water of Lake Geneva across different seasons contribute to viral inactivation. These findings contribute to our understanding of the inactivation of human enteric viruses in natural waterbodies—a hitherto understudied ecosystem service.

## INTRODUCTION

Enteric viruses are ubiquitous contaminants of surface- and groundwater systems (1), but the public health risk exerted by waterborne viruses critically depends on their environmental stability (2, 3). Despite the importance of environmental stability for waterborne virus transmission, the current understanding of this trait is incomplete. While virus inactivation by abiotic stressors, such as sunlight or temperature, has been extensively investigated (4, 5), less is known about the role of microbial communities.

Microorganisms can both promote and lower the environmental stability of viruses. Stabilizing interactions include the binding of viruses to bacterial lipopolysaccharides and peptidoglycans, which can enhance the virus’ thermostability and infectivity, or the internalization of viruses by protists, which can shield viruses from environmental stressors (6). In freshwater systems, however, such stabilizing interactions are typically outcompeted by inactivating processes, leading to a decrease in infectious virus titers over time (7–11). Bacteria importantly contribute to microbially mediated inactivation (11), though the associated kinetics and mechanisms are not well understood and appear to be highly organism specific. For example, isolates of the marine bacteria *Moraxella* were found to exhibit a virucidal effect on poliovirus, while no inactivation was observed for other enteric viruses (12). Similarly, many bacterial isolates from Lake Geneva were found to readily inactivate coxsackievirus A9, while inactivation of echovirus 11 was limited to fewer strains (13). Virus inactivation by bacteria is thought to be mediated by extracellular enzymes released into the environment. For instance, the inactivation of various members of the *Enterovirus* genus in freshwater could be linked to the activity of proteolytic enzymes, specifically serine proteases and matrix metalloproteases (MMPs) (13–15). Strikingly, however, the antiviral effect of proteases appears to be selective. For example, pronase was found to inactivate coxsackieviruses A7, A9 and B1, but not coxsackieviruses B1 and B3, nor poliovirus types 1–3. The serine protease trypsin was even more specific and only targeted coxsackievirus A9 (14). Similarly, the serine protease subtilisin A was found to inactivate coxsackievirus A9, but not echovirus 11 or coxsackievirus B5 (16).

Given the specificity of antiviral effects exerted by bacteria, it is apparent that bacterial inactivation of viruses in a given water body will likely depend on the composition of the indigenous microbial community. Yet, most of the work, so far, has addressed bacterially mediated virus inactivation using individual proteases or bacterial strains grown in isolation, whereas little is known about the importance of microbial diversity. On the one hand, the high specificity of enzymatically mediated inactivation indicates that diversity could be important. More diverse bacterial communities are more likely to include a member that produces a specific enzyme capable of inactivating a specific viral species. On the other hand, the production of extracellular enzymes is energetically costly, but benefits that arise from interactions with other microbes and shared (public) goods can surpass these costs (17). Hence, in more diverse communities, more such interactions can occur, stabilizing the production of extracellular enzymes that may lead to the inactivation of human enteric viruses.

The main objective of this study was to investigate how lake bacterial diversity influences the inactivation of enteric viruses. We frame this study in light of research on diversity–ecosystem service relationships. Multiple recent studies have highlighted the importance of microbial diversity on various ecosystem functions and services (18–22), and metastudies concluded that richness (i.e., the number of taxa) is generally positively associated with the performance of ecosystem functions and services (17, 23, 24). However, the inactivation of human enteric viruses represents a hitherto underappreciated ecosystem service.

There are several possible shapes of diversity–function relationships, including linear, log-linear, and saturating shapes, with positive-saturating relationships, consistent with theoretical expectations, being the most common (25). Better understanding of such relationships is important, as, for example, saturating relationships can reflect the presence of functionally redundant taxa, or idiosyncratic relationships may reflect the importance of keystone taxa. However, it is often not trivial to experimentally assess the shape of these associations due to the limited range in species richness (26) particularly in microbial communities when few isolated strains are combined to obtain diversity gradients. Dilution-to-extinction represents an alternative experimental approach that can overcome some of these limitations (e.g., references 21, 27). Basically, aquatic bacterial diversity can readily be manipulated by serially diluting a natural community and re-growing these cultures in sterile lake water medium. In this process, initially rare (i.e., low relative abundance) taxa are gradually removed, and the most diluted cultures (i.e., lowest diversity) represent the initially most abundant community members.

Given the high apparent specificity of virus inactivation, we hypothesize that this ecosystem service may be associated with rare lake water bacteria; hence, we expect that more diluted cultures would show reduced inactivation. To address this, we performed repeated dilution-to-extinction experiments using natural bacterial communities sampled from Lake Geneva and assessed the kinetics of echovirus 11 inactivation and genome decay in relation to bacterial diversity. We then identified taxa and species whose presence is associated with high inactivation rates and may thus serve as drivers of virus inactivation. Overall, our findings provide a first appreciation of the importance of diversity for bacterial-induced human virus inactivation in lakes and point toward the relevance of rare bacterial community members.

## MATERIALS AND METHODS

### Virus propagation and enumeration

We use human echovirus 11, as member of the *Enterovirus* genus, as a representative of waterborne enteric viruses. Echovirus 11 (Gregory strain, ATCC VR737) stock was prepared by infecting sub-confluent layers of Buffalo green monkey kidney cells (BGMK; kindly provided by Spiez Laboratory, Switzerland) as described in reference 28. In brief, cells were grown and maintained in minimum essential medium (ThermoFisher Scientific), with 1% penicillin–streptomycin (Gibco, Frederick, MD) and amended with 10% (growth medium) or 2% (maintenance medium) fetal bovine serum (ThermoFisher Scientific), at 37°C in 5% CO_2_. Sub-confluent cell monolayers were infected with echovirus 11, and viruses were propagated for 3 days. Subsequently, the viruses were released from cells by freeze–thawing three times. Cell debris was eliminated by centrifugation at 3,500 rpm for 5 min.

Infectious echovirus 11 was enumerated using a most probable number (MPN) approach as described previously (28). Briefly, five replicates of each sample were 10-fold serially diluted in maintenance medium and added to 95% confluent BGMK cells on a 96-well plate (Greiner CELLSTARVR 96-well plates; Sigma Aldrich). The plates were incubated at 37°C in 5% CO_2_ for 5 days. Each well was then examined for the occurrence of a cytopathic effect (CPE) by inverted microscopy. The number of CPE-positive wells was converted to most probable number per mL (MPN/mL) using the R package MPN. Samples were considered as quantifiable if at least one of the five wells containing the lowest dilution of a given sample yielded a positive CPE.

Echovirus 11 genomes were enumerated by reverse transcription digital PCR (RT-dPCR), targeting a 196-base amplicon in the 5′ untranslated region. This amplicon covers only a small fraction of the entire viral genome (7.4 kilobases) and does not represent the entirety of the viral genome. However, it is reasonable to assume that in microbially active systems, the entire RNA is rapidly degraded as soon as it is accessible to microbial RNAses. We, therefore, assume that the degradation of the 5′UTR coincides with the degradation of the entire viral genome. Viral RNA was extracted from samples using the QIAamp viral RNA Mini Kit (Qiagen) according to the manufacturer’s protocol. The extracted RNA was stored at −20°C prior to quantification. An RT-dPCR assay for enterovirus (EV) RNA quantification was optimized by adapting the thermal cycling conditions, primers, and probe concentrations from a previously described RT-qPCR assay (29–32). Hundred-fold diluted extracts were used as RNA templates. The assay was performed on a the QIAcuity One, 2-plex Device (Qiagen) using 8.5k 96-well Nanoplates (Qiagen) and the QIAcuity OneStep Advanced Probe Kit (Qiagen). The reaction volume was 12 µL and consisted of 3 µL of 4× OneStep Advanced Probe Master Mix, 0.12 µL of OneStep RT Mix, 1.5 µL of GC Enhancer, 1,000 nM forward primer (EV: 5′-CCTCCGGCCCCTGAATG-3′), 1,000 nM reverse primer (EV: 5′- ACCGGATGGCCAATCCAA-3′), 500 nM probe (EV: 5′-HEX-CGGAACCGACTACTTTGGGTGTCCGT-BHQ1-3′) (Microsynth, Switzerland), 3 or 4 µL of template RNA, and nuclease-free water. The PCR program included an RT step at 50°C for 40 min, 95°C for 2 min for enzyme activation, followed by 45 cycles of denaturation (95°C for 15 s) and annealing/extension (at 60°C for 1 min). In each run, a negative control (no template) and a positive control (echovirus 11 stock solution) were included. Data were analyzed using QIAcuity Software Suite 2.1.7.182 (QIAGEN). Quantities were expressed as genome copies per mL of sample (GC/mL). The RT-dPCR assays were performed using automatic settings for the threshold and baseline.

### Sample collection and culturing

Lake water samples were taken across three seasons (winter, summer, and fall) in 2023 from Lake Geneva. Specifically, for the winter sample, 10 L of lake water was collected on 5 January at a depth of 1.5 m from the LéXPLORE platform (https://lexplore.info/) located 570 m from the shore of Pully. The summer and fall samples were collected on 14 August and 21 November at a depth of approximately 1 m from the shore in Saint-Sulpice, Switzerland. Lake water was directly transported to the laboratory and stored at 4°C until processing. Sterile lake water was used as a cultivation medium and prepared by serially filtering through 0.8 µm of GF/D glass microfiber filters (Cytiva) and 0.22 µm of polycarbonate track-etched filters (Sartorius, Göttingen, Germany) followed by autoclaving at 121°C for 15 min. To prepare the inocula, lake water was filtered through 0.8 µm of GF/D glass microfiber filters (Cytiva, Marlborough, MA, USA) to remove phytoplankton and eukaryotic predators.

Prior to inoculation, bacterial diversity was manipulated using a dilution-to-extinction approach. This method manipulates the diversity of communities by removing rare (i.e., low abundance) species first and retaining the most abundant species in the most diluted samples. For this, the pre-filtered (0.8 µm) lake water was serially diluted using sterile lake water medium. The resulting cultures were then allowed to regrow, and bacterial abundance was monitored using flow cytometry. Once the cultures reached approximately 10^6^ cells mL^−1^ (Fig. S1), a subsample of each culture was used to estimate echovirus 11 inactivation, while another subsample was used for characterization of bacterial diversity using 16S rRNA gene amplicon sequencing.

The range of dilutions, replicates, and regrowth times differed for each experiment. We adapted the dilution steps between experiments based on insights from the previous experiments—with the ultimate aim of covering as large a diversity gradient as possible. In winter, there were six dilutions without replicates: undiluted, 1:10, 1:100, 1:1,000, 1:10,000, 1:100,000, and a control (sterile lake water). The regrowth time was 21 days (i.e., time required to reach 10^6^ cells mL^−1^). In summer, we included three dilutions: undiluted (five replicates), 1:5 (five replicates), 1:10 (nine replicates), and a sterile control (triplicates). In fall, there were six dilutions: undiluted (four replicates) and 1:25 (four replicates), 1:50 (three replicates), 1:100 (three replicates), 1:250 (three replicates), 1:1,000 (three replicates) and a sterile control (three replicates). The regrowth time for summer and fall campaigns was 10 days. Overall, our experiments thus included 52 individual cultures. The cultures were incubated at room temperature (22°C ± 1°C) on a shaking table.

To monitor bacterial growth in these cultures, 0.9 mL of each culture was sampled daily/approximately every 24 h and fixed with 0.1 mL of 37% filtered formaldehyde (0.22 µm) and stored at 4°C until analysis. The number of bacteria was quantified using a volumetric flow cytometer Attune CytPix (ThermoFisher Scientific, Waltham, MS, USA). For this, 198 µL of the fixed sample was stained with 2 µL of 20 µM Syto13 nucleic acid stain (ThermoFisher Scientific) followed by incubation in the dark at room temperature for 15 min. The bacterial cells were identified using green fluorescence and side scatter properties. Finally, cell counts were adjusted accounting for fixative and nucleic acid stain used during sample preparation.

At the end of each regrowth period (i.e., when cell abundances reached ~10^6^ cells mL^−1^), each sample was split into two aliquots: one for characterization of the bacterial communities by amplicon sequencing and one for monitoring echovirus 11 decay.

### Amplicon sequencing of bacterial communities

After regrowth (i.e., when cell abundances reached ~10^6^ cells mL^−1^), 240 mL of each culture was vacuum filtered onto 0.22 µm of polycarbonate track-etched filters (Sartorius) and stored at −20°C. Subsequently, bacterial DNA was extracted using the DNeasy PowerWater Kit (Qiagen, Hilden, Germany) according to the manufacturer’s instructions. Extraction blanks (extraction following the manufacturer’s protocol without any input material) were included in every run. DNA extracts were quantified using Qubit and the dsDNA HS Assay (ThermoFisher Scientific) and checked for purity using a microvolume spectrophotometer (NanoDrop, ThermoFisher Scientific).

DNA extracts and blanks were then used for 16S rRNA gene amplification using the 16S Barcoding Kit SQK-RAB204 (Oxford Nanopore Technologies, Oxford, UK). The PCR reaction was carried out in a 50-µL volume containing 10 ng of input DNA, 25 µL of LongAmp Taq 2× master mix (New England Biolabs, Ipswich, MS, USA), 14 µL of nuclease-free water (ThermoFisher Scientific), and 1 µL of barcoded 27F (5′-ATCGCCTACCGTGAC-barcode-AGAGTTTGATCMTGGCTCAG-3′) and 1492R (5′-ATCGCCTACCGTGAC-barcode-CGGTTACCTTGTTACGACTT-3′) primer pair. The PCR conditions were as follows: initial denaturation at 95°C for 1 min, 25 cycles of 95°C for 20 s, 55°C for 30 s, 65°C for 2 min with a final extension at 65°C for 5 min. Subsequently, each PCR product was purified using Agencourt AMPure XP beads (Beckman Coulter, Brea, CA, USA) and quantified using Qubit. Then, barcoded libraries were pooled to an amount of 80 fmol in 10 µL of 10 mM Tris-HCl pH 8.0 with 50 mM NaCl. The pooled library was loaded into the FLO-MIN106 flow cell (Oxford Nanopore Technologies) and primed using Flow Cell Priming Kit EXP-FLP002 (Oxford Nanopore Technologies) according to the manufacturer’s protocol. The MinION Mk1C sequencing device was run for up to 17 h. The sequence data were basecalled real time using the minKNOW software (v23.04.5, Oxford Nanopore Technologies). FASTQ files were used to estimate relative abundance and assign taxonomy to the species level using the cloud-based EPI2ME (Oxford Nanopore Technologies) workflow using the Kraken classifier. Bacterial species detected in extraction blanks were removed from the species abundance table using the function *decon* from the R package microDecon (McKnight et al. 2019). The function was applied separately on read count data for each sequencing run. After decontamination, bacterial taxa that were found once or had only had one read across samples in each experiment were removed. Rarefaction curves were constructed for each experiment using the *rarecurve* function in the R package vegan (Oksanen et al. 2024). Sequencing depth differed across experiments, ranging from 24 to 495,597 reads per sample. The abundance tables from the three experiments were merged and rarefied using the *rrarefy* function from the R package vegan (Oksanen et al. 2024). The rarefaction threshold was set at 105,000 reads per sample. After rarefaction, 17 of the 52 samples were excluded from further analysis, such that 35 samples remained for inclusion in the final data set (winter *n* = 6, summer *n* = 12, and fall *n* = 17).

### Echovirus 11 decay experiments

Regrown lake water samples were spiked with echovirus 11 and monitored for both infectivity loss and genome decay. For the winter samples, 100 mL of lake water culture was amended with echovirus 11 to a concentration of 10^10^ MPN/mL. For summer and fall experiments, 10 mL of lake water culture was amended with echovirus 11 to a concentration of 10^6^ MPN/mL. All spiked samples were incubated at room temperature on a shaking table and monitored over 96 h. One-milliliter aliquots were taken every 24 h and stored at −20°C until enumeration. The residual virus concentration in each sample was determined by both MPN assay and RT-dPCR.

### Data analysis

Microbial community analyses were performed using the *microeco* R package (33). Observed taxonomic richness (i.e. number of species) was used as alpha diversity estimate. We further estimated diversity (i.e., including estimates of undetected bacterial species) using the bias-corrected Chao-1 predictor implemented in the *estimateR* function of vegan. To investigate compositional changes across seasons and dilutions, we used principal coordinate analysis (PCoA) of Bray–Curtis similarities using the R function *cmdscale*.

Echovirus 11 inactivation rate constants were determined by fitting a first-order decay model to the decay curve (equation 1).


(equation 1) $$ln(\frac{C}{C_{0}})=-k_{i}t$$

where *C* and *C*_0_ (in MPN/mL) are infectious virus concentrations at time *t* and *t*_0_ respectively, and *k_i_* is the inactivation rate constant (h^−1^). In the winter samples, the *t*_0_ samples yielded unreliable results due to a difficulty in enumerating CPE. For these samples, we therefore used data measured at 24, 72, and 96 h and back-extrapolated *t*_0_. Extrapolated values corresponded well with our expectations based on dilution of the echovirus 11 stock used in this experiment.

Genome decay curves exhibited an initial lag phase; therefore, the first-order model fit to determine the genome decay rate constant was only applied to data points starting from 48 h (equation 2):


(equation 2) $$ln(\frac{N}{N_{48}})=-k_{g}t$$

where *N* and *N_48_* are the genome concentration (in GC/mL) at times *t* and 48 h, respectively, and *k_g_* is the genome decay rate constant (h^−1^).

To unravel the strength and shape of diversity–echovirus 11 inactivation relationships, we used Spearman rank correlations (function *cor.test* in R), linear models (function *lm* in R), and self-starting logistic non-linear least square models (function *nls* in R). More specifically, we used the self-starting function (*SSlogis* in R) to fit an asymptote, an inflection point, and a scale parameter for the non-linear model.

Linear discriminant analysis effect size (LEfSe) (34) was used to identify bacterial species (i.e., biomarkers) and higher taxonomic levels (genera, families) related to echovirus inactivation. For this, samples were binned into approximately equally sized bins of low (<0.036 h^−1^, *n* = 10), low-intermediate (0.038–0.068 h^−1^, *n* = 8), intermediate-high (0.069–0.086 h^−1^, *n* = 8), and high (> 0.086 h^−1^, *n* = 9) inactivation rate constants.

If not indicated otherwise, we use average ± standard deviation of replicate incubations to report results throughout the text.

## RESULTS

### Bacterial community composition and diversity

**Fig 1:**
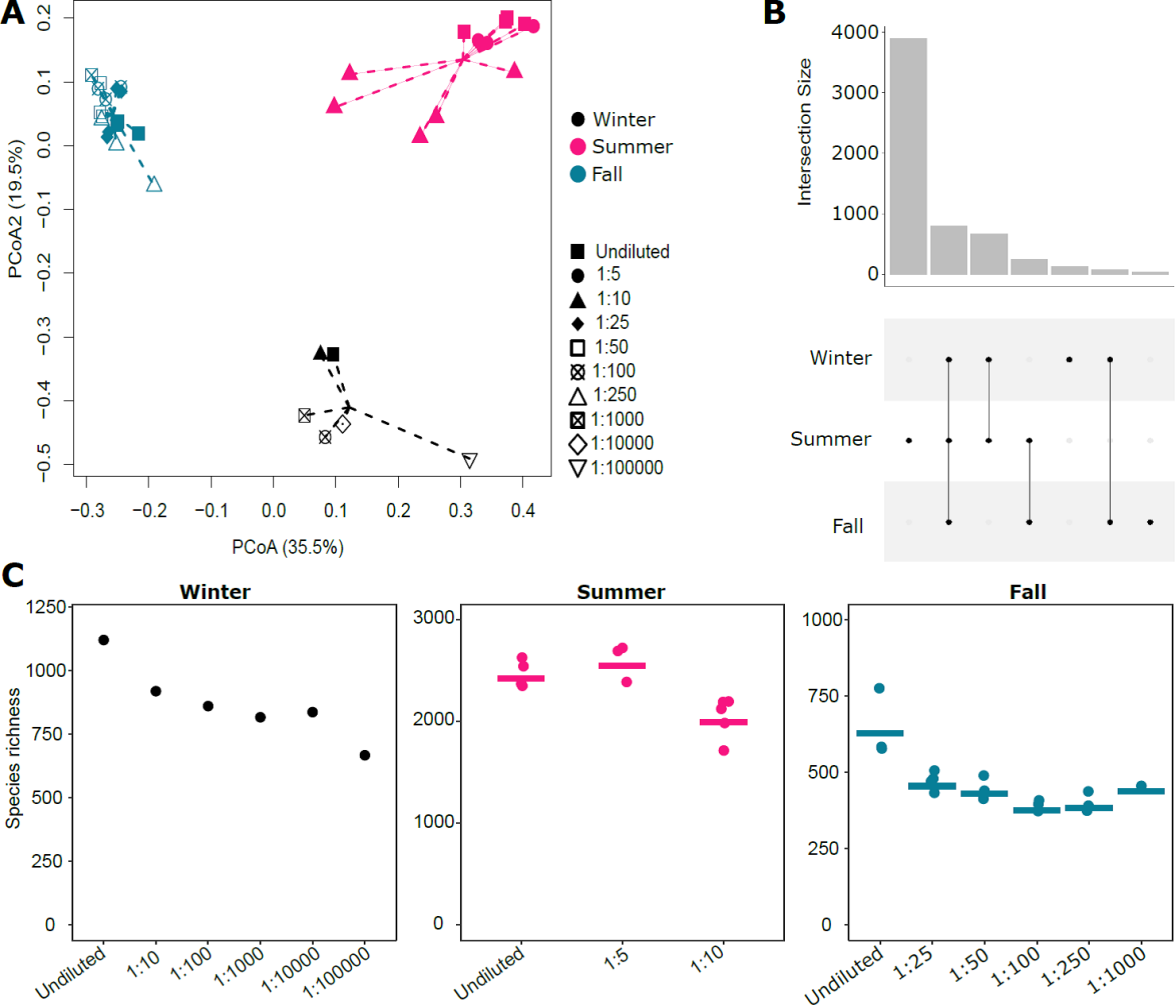
(**A**) Principal coordinate analysis of bacterial communities based on Bray–Curtis distance matrix. This ordination plot depicts 55% of the variation in community similarity across samples obtained during the different seasons. Note that seasonal turnover is much more pronounced than the effect of dilutions. (**B**) An upset plot shows the number of commonly and uniquely detected bacterial species across different experiments. (**C**) Bacterial species richness in lake water cultures of different dilutions is shown for the different seasons. Horizontal lines in summer and fall indicate average bacterial species richness in these samples.

We analyzed diversity in samples taken during three different seasons (summer *n* = 12, fall *n* = 17, and winter *n* = 6) and focused on richness (i.e., the number of bacterial taxa with species-level taxonomic assignment based on full-length 16S rRNA gene sequences; hereafter, we refer to this as “species richness”) as a metric of alpha diversity. Overall, we retained 5,897 bacterial species across all samples and seasons. Most species (range between 583 and 2,628 species) were detected in undiluted lake water incubations of which replicates obtained in summer exhibited the highest richness (from 2,349 to 2,628 species), followed by incubations in winter (1,114 species) and fall (from 578 to 775 species). Chao1 estimates of richness in these samples ranged between 719 and 3,590 species, suggesting that most (on average: 78.4 ± 4.7%) of the expected richness was detected with our sequencing effort. Across the three seasons, 800 species were commonly found and represented the putative lake water core community (Fig. 1). Members of the core accounted on average for 74.8% of relative abundance, whereas species detected in a single season accounted on average for 7.7%, and species detected across two seasons accounted for 17.5% of relative abundance. Samples taken in summer had the highest number of uniquely found species (*n* = 3,895), followed by winter (*n* = 140) and fall (*n* = 44). Members of Comamonadaceae (relative abundance ranging between 31.8% in winter and 91.3% in fall), Burkholderiaceae (1.4%–23.9%), Cytophagaceae (0.23%–7.1%), Sphingomonadaceae (0.1%–4.2%), and Pelagibacteraceae (0.4%–7.1%) dominated the bacterial communities across all seasons (Fig. S2). In total, we detected 2,380 bacterial genera, of which *Limnohabitans* (five species, relative abundance range between 13.1% and 56.3%), *Polaromonas* (eight species, 2.2%–11.6%), *Limnobacter* (three species, 0.0%–16.6%), and *Polynucleobacter* (10 species, 0.2%–6.5%) characterized the communities.

Despite the consistent taxonomic core, principal coordinate analysis based on Bray–Curtis dissimilarity (i.e., accounting for relative abundance of species) revealed substantial turnover in communities sampled during different seasons (Fig. 1). Given that more diluted samples are expected to represent a subset of species of less diluted samples, it is not surprising that diluted samples were similar to undiluted samples of the same season. More specifically, the average Bray–Curtis similarities of samples obtained during the same season varied between 0.52 ± 0.22 in summer, 0.55 ± 0.29 in winter, and 0.70 ± 0.14 in fall. In contrast, the average Bray–Curtis similarities were much lower when samples of different seasons were compared, ranging between 0.22 ± 0.08 (summer vs winter samples) to 0.28 ± 0.06 (fall vs summer samples). Dilution-to-extinction sequentially removes rare (i.e., low abundance) species from cultures, resulting in gradients of diversity. Indeed, we found a gradual reduction in the number of species in experiments conducted in winter and fall, whereas a 1:5 dilution did not result in a reduction in richness in summer (Fig. 1). However, consistently across seasons, bacterial species richness in samples with dilutions larger than 1:10 were reduced compared to the undiluted controls (*t*-test, *t*_summer_ = 3.64, *P*_summer_ = 0.008; *t*_fall_ = 5.84, *P*_fall_ <0.001; no replicated cultures were obtained in winter). The gradual reduction in diversity was consistent among replicated cultures, as indicated by coefficients of variation ranging between 5.4% and 9.8% in summer and between 4.5% and 17.4% in fall. Comparable to the decrease in richness with increasing dilutions, we observed that similarity decreased as well. Compared to the undiluted treatments, Bray–Curtis similarities decreased from 0.62 to 0.27 along the dilution gradient in winter, from 0.91 to 0.73 in fall, and from 0.71 to 0.32 in summer (Fig. S3).

### Echovirus 11 inactivation and genome decay

**Fig 2:**
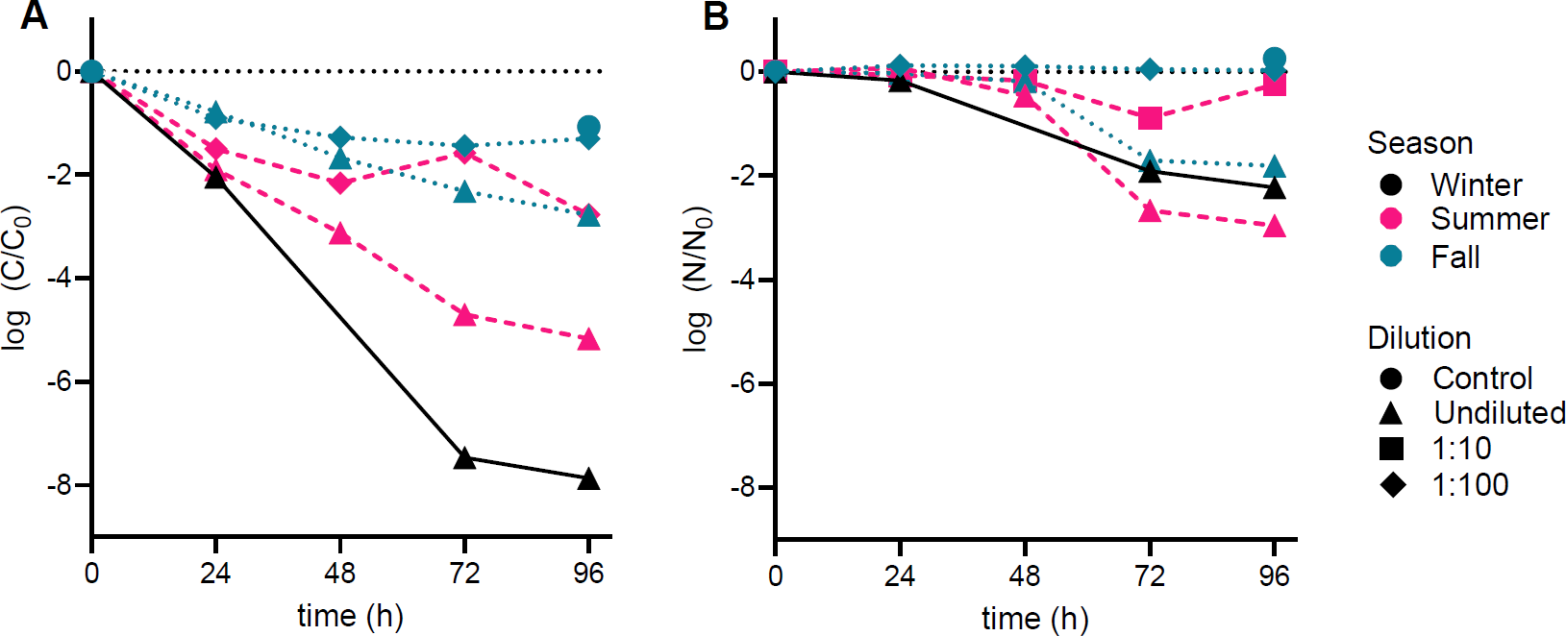
(**A**) Displayed is a subset of echovirus 11 inactivation data for different seasons and dilutions measured as loss of infectivity and (**B**) genome decay. Data of all infectivity and genome decay curves are displayed in Fig. S4 and S5.

We used two methods to quantify echovirus 11 decay in our samples. An MPN infectivity assay using a mammalian cell line allowed us to estimate the number of remaining infectious viruses (i.e., inactivation). Digital PCR, on the other hand, was used to monitor viral genome decay. Compared to sterile controls, echovirus 11 inactivation was always greater in samples including living bacteria (Fig. 2A; Fig. S4). While infectivity in controls was reduced by 1.1 to 3.0 log_10_ fold over 4 days, infectivity was reduced between 2.7 and 7.9 log_10_ fold in undiluted cultures. Across all seasons, the highest inactivation was observed in undiluted samples (winter: 7.9 log_10_ fold, summer: 5.2 log_10_ fold, and fall: 3.0 log_10_ fold). Accordingly, inactivation rate constants were the lowest for controls (*k*_*i*_ = 0.03 ± 0.01 h^−1^) and highest for undiluted samples (winter: *k*_*i*_ = 0.19 h^−1^, summer: *k*_*i*_ = 0.09 ± 0.01 h^−1^, and fall: *k*_*i*_ = 0.07 ± 0.003 h^−1^).

Compared to loss of infectivity, genome decay was generally lower. Particularly for the first 24 h, dPCR revealed no changes in genome integrity, suggesting a lag phase (Fig. 2B; Fig. S5). However, similar to inactivation, genome decay remained low in autoclaved controls (ranging between −0.3 and 0.4 log_10_ fold over 96 h) and was the highest in undiluted samples ranging between 1.2 and 3.0 log_10_ fold reductions. Genome decay rate constants, accounting for the initial lag phase, differed across seasons with the highest rates obtained in undiluted samples in summer (*k*_*g*_ = 0.10 ± 0.01 h^−1^), followed by fall (*k*_*g*_ = 0.08 ± 0.008 h^−1^) and winter (*k*_*g*_ = 0.03 h^−1^).

Dilution of bacterial communities often led to a reduction in inactivation compared to undiluted samples (Fig. S6). For instance, compared to the 7.9 log_10_ fold reduction in echovirus titers in undiluted samples, infectivity in 1:10 diluted samples was reduced to 6.6 log_10_ fold in winter. Similar reductions in inactivation were also observed in summer (between 3.7 and 5.2 log_10_ fold in undiluted samples and between 2.8 and 4.1 log_10_ fold in 1:10 diluted samples) and in fall (between 2.8 and 3.0 log_10_ fold in undiluted samples and between 1.3 and 1.9 log_10_ fold in 1:25 diluted samples). However, in summer, differences in inactivation in the 1:10 dilutions and inactivation in the undiluted samples were not statistically significantly (*t*-test, *t* = 1.91, *P* = 0.09), whereas in fall, inactivation in the 1:25 diluted samples was significantly lower compared to that in the undiluted samples (*t*-test, *t* = 5.83, *P* = 0.002).

Similarly, the magnitude of genome decay over 96 h in diluted samples was generally lower than in undiluted samples. In summer, this difference was statistically significant for 1:10 dilutions (genome decay in undiluted samples ranged between 2.0 and 3.0 log_10_ fold; genome decay in 1:10 diluted samples ranged between 0.3 and 0.8 log_10_ fold; *t*-test, *t* = 8.28, *P* < 0.001). In fall, genome decay was significantly reduced in 1:25 dilutions (genome decay in undiluted samples ranged between 1.2 and 2.3 log_10_ fold, genome decay in 1:25 diluted samples ranged between 0.9 and 1.7 log_10_ fold; *t*-test, *t* = 5.55, *P* = 0.003).

### Diversity–function relationship

**Fig 3:**
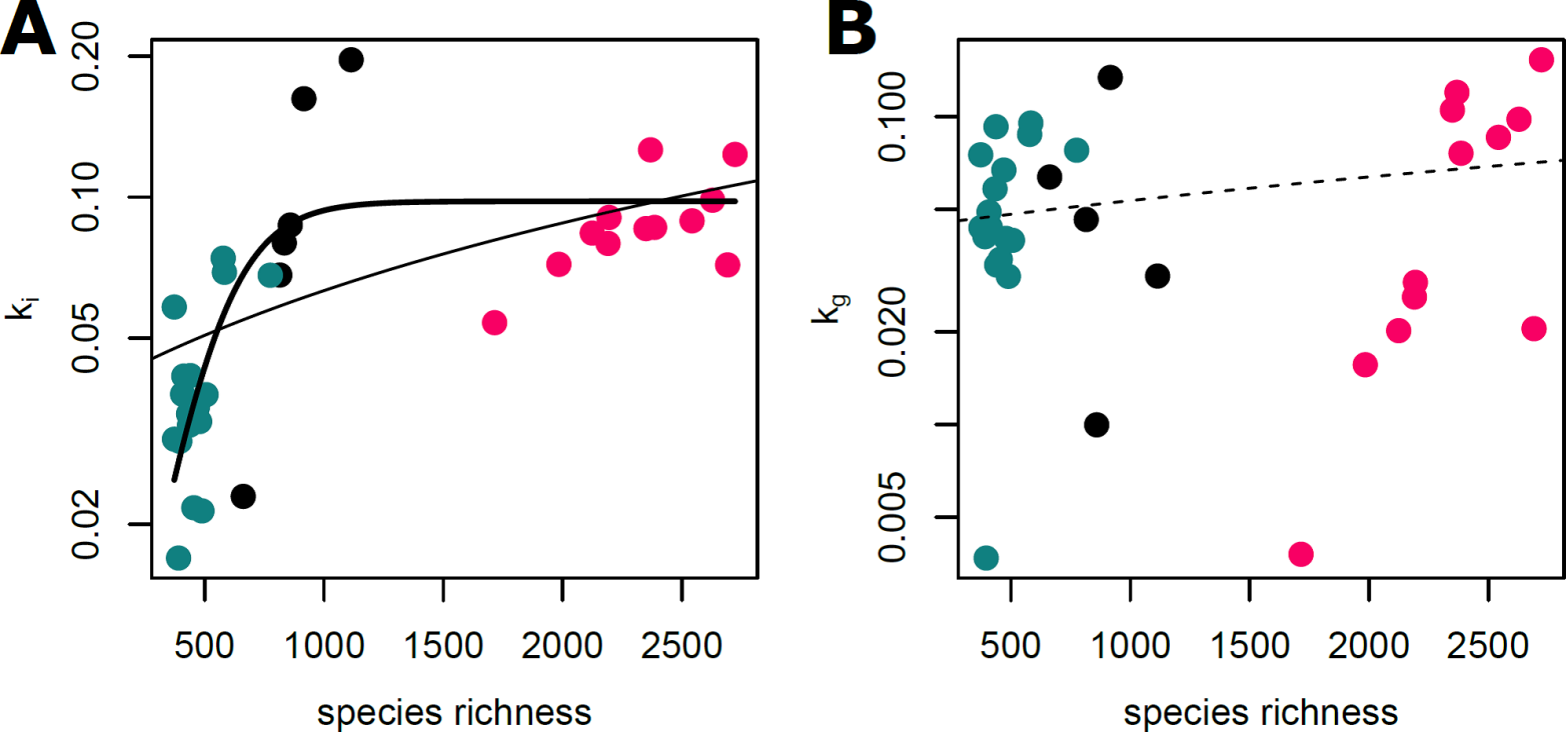
(**A**) Association between bacterial species richness and inactivation rate constants for infectivity loss (*k*_*i*_) and (**B**) genome decay (*k*_*g*_). The logistic and linear model fits are displayed as solid lines for the relationship between diversity and rate of loss of infectivity, while the dashed line represents the linear trend for genome decay (i.e., the linear model was non-significant). Symbol colors depict the different seasons (same as in Fig. 1).

To assess the relationship between bacterial diversity and virus inactivation as an ecosystem service, we correlated observed species richness with inactivation as well as with genome decay rate constants (Fig. 3). When the associations were tested for individual seasons, positive relationships between richness and inactivation rate constants were identified in all seasons. The rank correlation was significant in winter (Spearman rho = 1, *P* = 0.003), but marginally non-significant in summer (rho = 0.55, *P* = 0.06) and fall (rho = 0.39, *P* = 0.12). Similarly, the correlation between richness and genome decay rate constants was significant in summer (rho = 0.70, *P* = 0.01), but not in fall (rho = 0.21, *P* = 0.41) or winter (rho = −0.08, *P* = 0.91).

Combining the three experiments provided a larger diversity gradient, ranging from 373 to 2,722 bacterial species and revealed a positive relationship between inactivation and species richness (rho = 0.79, *P* < 0.001), whereas genome decay was not significantly related to species richness (rho = 0.12, *P* = 0.49). Compared to linear relationships between species richness and inactivation rate constants (Akaike Information Criterion [AIC] = −132), a logistic model (i.e., a non-linear model including an asymptote) better explained this relationship (AIC = −145).

Next, to incorporate the importance of species abundance in our diversity estimates, we tested the association between inactivation and genome decay rates with Shannon *H*′ and Simpson diversity indices. The association between the Shannon index and inactivation rates was positive and significant (rho = 0.50, *P* < 0.01) when samples of all seasons were combined. However, when considered individually, the associations were non-significant for all seasons. Similarly, the correlation between the Simpson Index and the inactivation rate constant was positive and significant (rho = 0.42, *P* = 0.01), while individual associations were not significant. Neither Shannon index nor Simpson index yielded significant correlations with genome decay rate constants. Both Shannon and Simpson indices are part of the Hill number (also called effective number of species) family of diversity estimates, which differ by their order *q*. An increase in order *q* increasingly emphasizes abundant species and discounts rare species (*q* = 0 returns species richness, *q* = 1 returns Shannon *H*, and *q* = 2 returns the inverse Simpson). We explored how the correlation between inactivation rate and genome decay constants changed along an increasing order *q* (Fig. S7). Considering all species present (i.e., *q* = 0) yielded a strong (Pearson correlation coefficient *r* = 0.54) and significant (*P* < 0.001) correlation with inactivation rate constant, and a weaker (*r* = 0.23) and non-significant (*P* = 0.18) relationship with genome decay rate constant. Gradually increasing the importance of abundant species (i.e., increasing order *q*) showed that the correlation with inactivation rate constant decreased to *r* = 0.31 and became non-significant at *q* > 1.5. Albeit non-significant, the correlation coefficients between diversity estimates of order *q* and genome decay rate constant also gradually decreased (reaching a minimum of *r* = 0.09).

### Identification of taxa correlated with high inactivation rates

**Fig 4:**
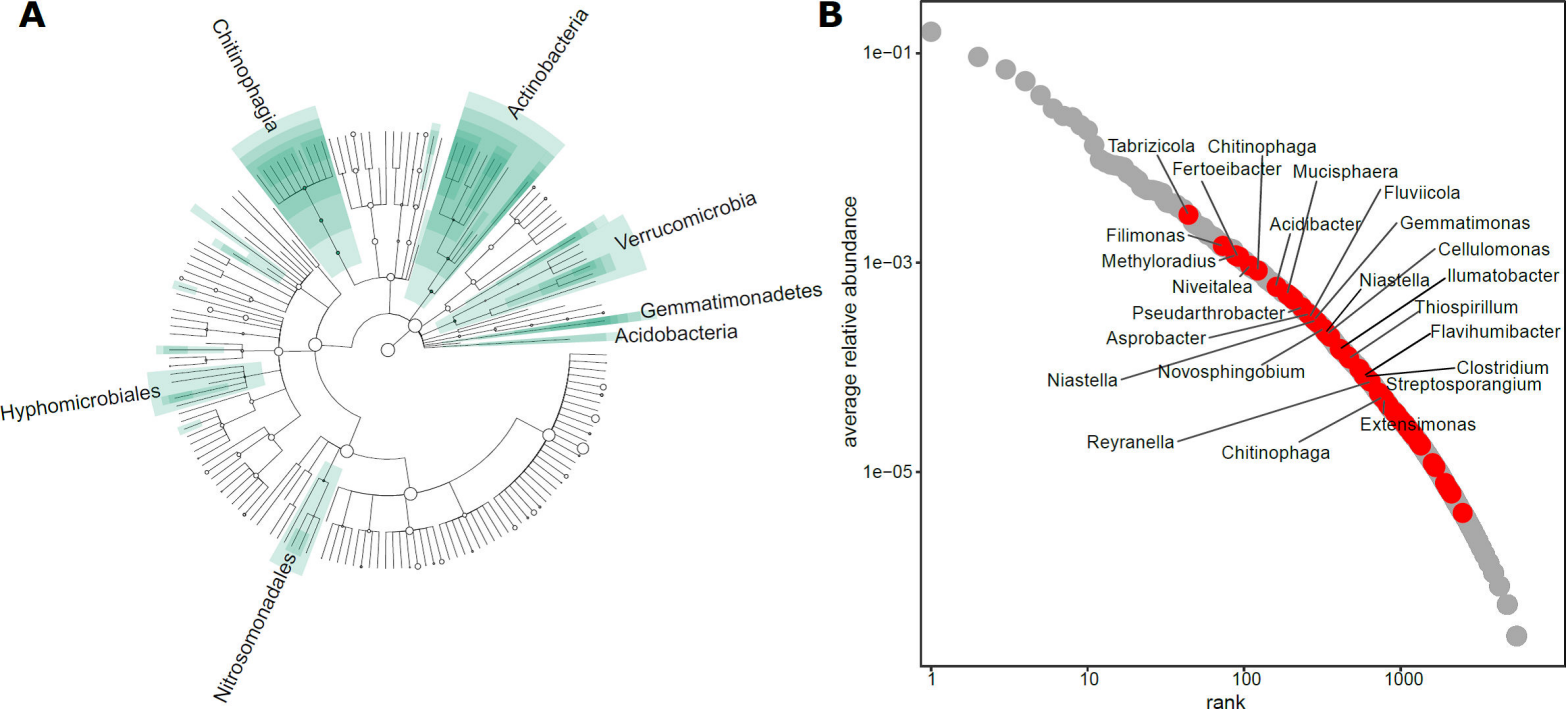
(**A**) Bacterial taxa related to echovirus 11 inactivation were identified using LEfSe. Results are depicted in a cladogram, which represents the taxonomic structure of the bacterial community. Circle size is proportional to the average relative abundance of the respective taxon. Taxonomies significantly related to high echovirus 11 inactivation rates are highlighted using green boxes. Higher-order taxonomies are highlighted using the respective taxon names. (**B**) The rank–abundance curve shows average bacterial species abundance distribution with species identified using LEfSe highlighted in red. Genus-level taxonomies are shown for selected biomarkers. Note that the 61 biomarkers of high echovirus 11 inactivation rate occurred at ranks above 44 (reflecting an average relative abundance of 0.3%) with a median rank of 583. A fully annotated version of this panel is available as Fig. S8.

Both the saturating relationship between diversity and virus inactivation rate constant (Fig. 3) and the correlation analysis along diversity order *q* (Fig. S7) suggest that rare (i.e. low abundance) bacterial species play an important role for viral inactivation in lake water. We used linear discriminant effect size analysis (LEfSe) to further pinpoint bacterial species whose abundance along the dilution-to-extinction series is associated with high inactivation rates (Fig. 4). LEfSe identified a total of 187 significant features. Strikingly, these features showed consistent taxonomies, suggesting that the mechanisms related to viral inactivation are taxonomically conserved. The most relevant and deep branching taxonomic entities related to high viral inactivation rates were *Acidobacteria*, *Gemmmatmonadetes*, *Verrumicrobia*, *Chitinophagia*, *Hyphomicrobiales*, and *Nitrosomonodales*. At the species level, a total of 61 bacterial species were found to be significantly associated with high inactivation rate constants (Table S1), most of them classified as Proteobacteria (*n* = 23), Bacteroidota (*n* = 19), and Actinobacteria (*n* = 14). Among them, the species classified as members of the *Chitinophagaceae* family were the most numerous (*n* = 17), highlighting the putative relevance of these common freshwater bacteria for enterovirus inactivation. Bacterial species with the highest linear discriminant analysis scores (i.e., >3) were *Tabrizicola oligotrophica*, *Filimonas zeae*, *Methyloradius palustris*, *Niveitalea solisilvae*, *Constrictibacter antarcticus*, *Chitinophaga eiseniae,* and *Fertoeibacter niger*. In line with our expectation that rare taxa are important for viral inactivation, these species had a relative abundance lower than 0.01 in undiluted samples. In terms of ranks (i.e., the on average most abundant species in our community obtains a rank of 1, the second most abundant community member obtains a rank of 2, etc.), biomarkers identified by LEfSe obtained ranks between 44 and 2,486.

## DISCUSSION

In line with earlier inactivation rate measures of echovirus 11 in lake water (11, 13), we observed pronounced echovirus 11 inactivation in different samples from Lake Geneva. Compared to the infectivity loss in undiluted samples (ranging between 2.7 and 7.9 log_10_ fold changes over 96 h across seasons), inactivation in sterile lake water was low (ranging between 1.1 and 3.0 log_10_ fold). This confirms that microorganisms are the main contributors to echovirus 11 in undiluted lake water samples, whereas abiotic inactivation is less important. Interestingly, we observed seasonal differences in echovirus 11 inactivation, with samples in winter showing the largest infectivity losses, followed by samples obtained in summer and fall, respectively. Such variation is striking because it indicates that seasonal changes in the lake water bacterial community (e.g. 35) have the potential to affect virus inactivation rates. We did not observe substantial season variation in viral inactivation in the sterile controls, suggesting that variation in abiotic factors (e.g., pH, hardness, nutrient availability) did not account for the observed seasonality. The influence of seasonal community turnover further points toward specific bacterial community members contributing to inactivation. This notion is supported by experimental measurements of echovirus 11 inactivation using 136 bacterial isolates from lake Geneva (13). There, echovirus 11 inactivation was restricted to <1 log_10_ over 48 h for most isolates, while only a few strains (i.e., classified as *Janthinobacterium*, *Chryseobacterium*, *Aeromonas*, and *Paraheinheimera*) were able to induce more than 2-log_10_ fold inactivation of echovirus 11. Hence, echovirus 11 inactivation seems to be an ecosystem function performed by specific freshwater bacterial community members.

Trends in echovirus 11 inactivation were contrasted by measurements of genome decay. In contrast to the instantaneous and exponential decay of infectious titers of echovirus 11, genome decay exhibited a 24-h lag phase. Moreover, genome decay was reduced by merely 1.2 to 3.0 log_10_ in undiluted lake water, substantially less than the corresponding loss of infectivity in these samples. These differences indicate that the primary target of microbially mediated echovirus 11 inactivation is the viral capsid, whereas genome damage is not a necessary condition for inactivation. This mechanism is consistent with previous work suggesting that bacterially produced proteases, specifically MMP and serine proteases may play a role in echovirus 11 inactivation (13), and that the interaction of viruses with these proteases ultimately leads to cleavage of the virus’ capsid proteins (16). Bacterial proteases may be released by bacteria into the environment to degrade proteins and peptides and aid in nutrient acquisition (36). Putatively as a side effect to their main role, the proteolytic activity of proteases produced by Lake Geneva bacteria may thus affect the structural integrity of echovirus 11 capsids, which may rapidly reduce echovirus 11 infectivity. This is supported by comparatively low activation energies required for echovirus inactivation, suggesting that only a few conformational changes are required for viral inactivation (37). The lag in genome decay suggests that it takes more time until the viral capsid is sufficiently cleaved, ultimately leading to the release or damage of viral RNA.

Alternatively, the discrepancy in infectivity and genome decay kinetics may arise from the internalization of viruses by protists or their adsorption to protists and bacteria. Such processes would render the infectious viruses difficult to enumerate and, hence, bias the infectivity data toward lower values. Here, however, we can neglect virus–protist interactions because protists were removed from our experimental solutions by filtration. Furthermore, in experiments with a structurally similar member of the *Enterovirus* genus (coxsackievirus B5; Fig. S9), no discrepancy in infectivity and genome decay kinetics was observed, indicating that the extent of virus adsorption to bacteria in our experimental system was negligible. This suggests that the observed inactivation kinetics reflects the proteolytic degradation of echovirus 11 capsids and is unbiased by internalization or adsorption processes.

In this context, better understanding of the role of diversity for viral inactivation is important. While microbes benefit from the activity of extracellular enzymes through the acquisition of energy and nutrients, enzyme production and excretion require a substantial resource and energy investment. In the absence of spatial structure, such as in pelagic systems, this represents a conundrum, as non-producing taxa also benefit from extracellular enzyme activity without sharing the cost of production (i.e., cheating). In diverse communities, this dilemma can be resolved by taxa interacting through multiple traits (38). Consequently, in more diverse communities, more interactions can occur, stabilizing the production of extracellular enzymes that may lead to the inactivation of human enteric viruses. Our results suggest a generally positive association between lake water bacterial diversity and echovirus 11 inactivation, highlighting the importance of diversity for this ecosystem service. These results are conceptually similar with findings of studies on the shape of the association of bacterial diversity with micropollutant biotransformation (39, 40). Enteric viruses are comparable to micropollutants because both are present at low concentrations in the environment, which prevents them from being utilized as a growth substrate. Furthermore, both are contaminants rather than natural constituents of ecosystems, such that bacteria likely did not evolve to metabolically degrade them. The typically low abundance of enteric viruses in unpolluted lake water may suggest that free-living bacteria do not rely on them as a carbon or nutrient source. However, cleavage of more abundant, naturally occurring viruses infecting bacteria (i.e., phages) can release nucleic acids, which are rich in phosphorus (41). Although not much is known about the mechanism of virovory, in oligotrophic systems, such as the pelagic zone of Lake Geneva, certain bacteria may indeed release extracellular enzymes to the environment to access the viral P pool. Hence, besides a side effect of extracellular enzymes produced by bacteria to decompose proteinaceous compounds, these enzymes may indeed be released to the environment to cleave viral capsids. Nevertheless, inactivation of human enteroviruses putatively remains a side effect of such phage-directed virovory.

Working on activated sludge, Johnson et al. (39) reported different shapes of diversity–function relationships to best explain the effects of biodiversity on the biotransformation of specific micropollutants. Similarly, our results suggested a generally positive influence of diversity on the rates of echovirus 11 inactivation and genome decay, but an asymptotic logistic model better explained the relationship for echovirus 11 inactivation rate. Such a model suggests that above a certain threshold diversity, additional diversity does not contribute to a further increase in function. Such saturating relationships are considered hallmarks of functional redundancy, reflecting the notion that specific functions are performed by multiple species. This is important as it suggests that a loss of diversity, to a certain extent, may not influence the rate of echovirus 11 inactivation in lake water, providing a buffer against natural fluctuation. However, a study using dilution-to-extinction to elucidate the shape of the relationship between bacterial diversity and the degradation of pollutants found non-saturating relationships in two freshwater ecosystems (42). In fact, when analyzing the relationship between diversity and echovirus 11 inactivation rate for each season individually, we could also not establish unanimous relationships. This may suggest that a large diversity gradient is required to resolve the saturating shape of diversity–echovirus 11 inactivation relationships. However, we cannot exclude that additional factors, such as seasonal variation in lake water nutrients or physiologic state of microbes, also contribute to this observation.

Our findings contribute to the idea that specialized ecosystem functions critically depend on biodiversity due to the importance of species with unique functional profiles. For example, specialized functions, such as sulfate, iron reduction, and degradation of complex substrates (phenanthrene, chitin, cellulose, carbon) are sensitive to a reduction in diversity (43). Here, the majority of identified bacteria associated with high inactivation of echovirus 11 belonged to phyla Bacteroidota and Proteobacteria. Two studies observed that *Pseudomonas* sp. induces inactivation, and among the identified bacterial species in our analysis, we also identified *Pseudomonas carboxydohydrogena*. These findings are consistent with the taxonomy of cultured isolates that were found to inactivate echovirus 11 (13, 14). In our analysis, we also identified bacteria from the family Chitinophagaceae with many species belonging to *Chitinophaga genera*. Chitinophagaceae are ubiquitous bacteria and known for the production of hydrolytic enzymes for chitin and cellulose degradation (44, 45). Besides the potential for carbohydrate degradation, they are also found to harbor the potential for the production of proteases. The genome of *Chitinophaga* sp. was found to encode for proteolytic enzymes (46). In another study, a *Chitinophaga* isolate was found to express metallocarboxypeptidase (47). Interestingly, metallocarboxypeptidases have been suggested for SARS-CoV-2 therapy (48).

We used dilution-to-extinction cultures to manipulate diversity in lake water cultures. We argue that this approach is particularly useful to identify the role of rare taxa for specialized ecosystem functions such as the inactivation of human enteric viruses. Rarity is a hallmark of bacterial communities and often linked to oligotrophic conditions, and rare taxa were found to be disproportionately important for specialized functions (49, 50). The gradual dilution in our experiments removed initially rare taxa first, retaining only the most abundant taxa in the most diluted cultures. In contrast to the assembly of isolates into co-cultures of varying diversity, this, therefore, allows inference of the importance of rare species. At the same time, we are aware of limitations of the study. First, in some instances, dilution was not sufficient to significantly reduce diversity. Moreover, in this study, we only investigated the effect of diversity on a single human virus, but similar experiments could be done for other (enteric) viruses. Such work may shed light on the importance of considering multiple functions simultaneously (i.e., multifunctionality). Future work based on the results of this study may also attempt to isolate some of the candidate bacterial species identified here and experimentally confirm the inactivation of echovirus 11 and identify enzymes involved in inactivation.

In summary, our findings demonstrate that the environmental stability of echovirus 11 in surface water decreases with increasing diversity of the bacterial community and that rare, but taxonomically well-constrained, species are associated with high inactivation rates. This implies that microbially diverse surface water ecosystems have a high capacity for self-purification after a contamination event, whereas this capacity is reduced in low-diversity systems. Promoting or maintaining a high bacterial diversity in surface waters may help reduce waterborne disease outbreaks among recreational water users and may thus be protective of public health.

## Data Availability

All data are available on Zenodo under https://doi.org/10.5281/zenodo.13642583. Sequence reads were deposited at NCBI’s Sequence Read Archive (SRA) under Bioproject ID PRJNA1190214.

## SUPPLEMENTAL MATERIAL

Supplemental material aem.02366-24-s0001.pdf Figures S1 to S9; Table S1.

ASM does not own the copyrights to Supplemental Material that may be linked to, or accessed through, an article. The authors have granted ASM a non-exclusive, world-wide license to publish the Supplemental Material files. Please contact the corresponding author directly for reuse.

## References

[1] Wyn-Jones AP, Sellwood J. 2001. Enteric viruses in the aquatic environment. J Appl Microbiol 91:945–962. doi:10.1046/j.1365-2672.2001.01470.x 11851802

[2] Li C, Sylvestre É, Fernandez-Cassi X, Julian TR, Kohn T. 2023. Waterborne virus transport and the associated risks in a large lake. Water Res 229:119437. doi:10.1016/j.watres.2022.119437 36476383

[3] Crank KC, Rocha-Melogno L, Clements E, Bibby K. 2024. Assessing the impact of pathogen decay on quantitative microbial risk assessment infection estimates. ACS EST Water 4:3789–3797. doi:10.1021/acsestwater.4c00100

[4] Bertrand I, Schijven JF, Sánchez G, Wyn-Jones P, Ottoson J, Morin T, Muscillo M, Verani M, Nasser A, de Roda Husman AM, Myrmel M, Sellwood J, Cook N, Gantzer C. 2012. The impact of temperature on the inactivation of enteric viruses in food and water: a review. J Appl Microbiol 112:1059–1074. doi:10.1111/j.1365-2672.2012.05267.x 22380614

[5] Nelson KL, Boehm AB, Davies-Colley RJ, Dodd MC, Kohn T, Linden KG, Liu Y, Maraccini PA, McNeill K, Mitch WA, Nguyen TH, Parker KM, Rodriguez RA, Sassoubre LM, Silverman AI, Wigginton KR, Zepp RG. 2018. Sunlight-mediated inactivation of health-relevant microorganisms in water: a review of mechanisms and modeling approaches. Environ Sci: Processes Impacts 20:1089–1122. doi:10.1039/C8EM00047F PMC7064263 30047962

[6] Zhang M, Altan-Bonnet N, Shen Y, Shuai D. 2022. Waterborne human pathogenic viruses in complex microbial communities: environmental implication on virus infectivity, persistence, and disinfection. Environ Sci Technol 56:5381–5389. doi:10.1021/acs.est.2c00233 35434991 PMC9073700

[7] Herrmann JE, Kostenbader KD Jr, CLIVER DO. 1974. Persistence of enteroviruses in lake water. Appl Microbiol 28:895–896. doi:10.1128/am.28.5.895-896.1974 4374125 PMC186847

[8] Sobsey MD, Shields PA, Hauchman FH, Hazard RL, Caton LW III. 1986. Survival and transport of hepatitis A virus in soils, groundwater and wastewater. Water Sci Technol 18:97–106. doi:10.2166/wst.1986.0116

[9] Yates MV, Stetzenbach LD, Gerba CP, Sinclair NA. 1990. The effect of indigenous bacteria on virus survival in ground water. J Environ Sci Health Part A: Environ Sci Eng Toxicol 25:81–100. doi:10.1080/10934529009375541

[10] Gordon C, Toze S. 2003. Influence of groundwater characteristics on the survival of enteric viruses. J Appl Microbiol 95:536–544. doi:10.1046/j.1365-2672.2003.02010.x 12911702

[11] Olive M, Gan C, Carratalà A, Kohn T. 2020. Control of waterborne human viruses by indigenous bacteria and protists is influenced by temperature, virus type, and microbial species. Appl Environ Microbiol 86:e01992-19. doi:10.1128/AEM.01992-19 31732569 PMC6974641

[12] Girones R, Jofre JT, Bosch A. 1989. Isolation of marine bacteria with antiviral properties. Can J Microbiol 35:1015–1021. doi:10.1139/m89-169 2558789

[13] Corre M-H, Bachmann V, Kohn T. 2022. Bacterial matrix metalloproteases and serine proteases contribute to the extra-host inactivation of enteroviruses in lake water. ISME J 16:1970–1979. doi:10.1038/s41396-022-01246-3 35545659 PMC9296489

[14] Cliver DO, Herrmann JE. 1972. Proteolytic and microbial inactivation of enteroviruses. Water Res 6:797–805. doi:10.1016/0043-1354(72)90032-2

[15] Ward RL, Knowlton DR, Winston PE. 1986. Mechanism of inactivation of enteric viruses in fresh water. Appl Environ Microbiol 52:450–459. doi:10.1128/aem.52.3.450-459.1986 3021056 PMC203555

[16] Corre M-H, Rey B, David SC, Torii S, Chiappe D, Kohn T. 2024. The early communication stages between serine proteases and enterovirus capsids in the race for viral disintegration. Commun Biol 7:969. doi:10.1038/s42003-024-06627-2 39122806 PMC11316004

[17] Lefcheck JS, Byrnes JEK, Isbell F, Gamfeldt L, Griffin JN, Eisenhauer N, Hensel MJS, Hector A, Cardinale BJ, Duffy JE. 2015. Biodiversity enhances ecosystem multifunctionality across trophic levels and habitats. Nat Commun 6:6936. doi:10.1038/ncomms7936 25907115 PMC4423209

[18] Reinthaler T, Winter C, Herndl GJ. 2005. Relationship between bacterioplankton richness, respiration, and production in the Southern North Sea. Appl Environ Microbiol 71:2260–2266. doi:10.1128/AEM.71.5.2260-2266.2005 15870310 PMC1087554

[19] Jiang L. 2007. Negative selection effects suppress relationships between bacterial diversity and ecosystem functioning. Ecology 88:1075–1085. doi:10.1890/06-1556 17536392

[20] Lindström ES, Feng XM, Granéli W, Kritzberg ES. 2010. The interplay between bacterial community composition and the environment determining function of inland water bacteria. Limnol Oceanogr 55:2052–2060. doi:10.4319/lo.2010.55.5.2052

[21] Peter H, Beier S, Bertilsson S, Lindström ES, Langenheder S, Tranvik LJ. 2011. Function-specific response to depletion of microbial diversity. ISME J 5:351–361. doi:10.1038/ismej.2010.119 20686511 PMC3105700

[22] Lyons MM, Dobbs FC. 2012. Differential utilization of carbon substrates by aggregate-associated and water-associated heterotrophic bacterial communities. Hydrobiologia 686:181–193. doi:10.1007/s10750-012-1010-7

[23] Balvanera P, Pfisterer AB, Buchmann N, He J-S, Nakashizuka T, Raffaelli D, Schmid B. 2006. Quantifying the evidence for biodiversity effects on ecosystem functioning and services. Ecol Lett 9:1146–1156. doi:10.1111/j.1461-0248.2006.00963.x 16972878

[24] Cardinale BJ, Srivastava DS, Duffy JE, Wright JP, Downing AL, Sankaran M, Jouseau C. 2006. Effects of biodiversity on the functioning of trophic groups and ecosystems. Nature New Biol 443:989–992. doi:10.1038/nature05202 17066035

[25] Schmid B, Balvanera P, Cardinale BJ, Godbold J, Pfisterer AB, Raffaelli D, Solan M, Srivastava DS. 2009. Edited by S. Naeem, D. E. Bunker, A. Hector, M. Loreau, and C. Perrings. Consequences of species loss for ecosystem functioning: meta-analyses of data from biodiversity experiments, p 14–29. Oxford University Press, Oxford, UK.

[26] Miki T, Yokokawa T, Matsui K. 2014. Biodiversity and multifunctionality in a microbial community: a novel theoretical approach to quantify functional redundancy. Proc R Soc B 281:20132498. doi:10.1098/rspb.2013.2498 PMC3871314 24352945

[27] Roger F, Bertilsson S, Langenheder S, Osman OA, Gamfeldt L. 2016. Effects of multiple dimensions of bacterial diversity on functioning, stability and multifunctionality. Ecology 97:2716–2728. doi:10.1002/ecy.1518 27859115

[28] Carratalà A, Shim H, Zhong Q, Bachmann V, Jensen JD, Kohn T. 2017. Experimental adaptation of human echovirus 11 to ultraviolet radiation leads to resistance to disinfection and ribavirin. Virus Evol 3:vex035. doi:10.1093/ve/vex035 29225923 PMC5714166

[29] Tsai YL, Sobsey MD, Sangermano LR, Palmer CJ. 1993. Simple method of concentrating enteroviruses and hepatitis A virus from sewage and ocean water for rapid detection by reverse transcriptase-polymerase chain reaction. Appl Environ Microbiol 59:3488–3491. doi:10.1128/aem.59.10.3488-3491.1993 7504433 PMC182480

[30] Monpoeho S, Dehée A, Mignotte B, Schwartzbrod L, Marechal V, Nicolas J-C, Billaudel S, Férré V. 2000. Quantification of enterovirus RNA in sludge samples using single tube real-time RT-PCR. Biotechniques 29:88–93. doi:10.2144/00291st03 10907082

[31] Kageyama T, Kojima S, Shinohara M, Uchida K, Fukushi S, Hoshino FB, Takeda N, Katayama K. 2003. Broadly reactive and highly sensitive assay for Norwalk-like viruses based on real-time quantitative reverse transcription-PCR. J Clin Microbiol 41:1548–1557. doi:10.1128/JCM.41.4.1548-1557.2003 12682144 PMC153860

[32] Loisy F, Atmar RL, Guillon P, Le Cann P, Pommepuy M, Le Guyader FS. 2005. Real-time RT-PCR for norovirus screening in shellfish. J Virol Methods 123:1–7. doi:10.1016/j.jviromet.2004.08.023 15582692

[33] Liu C, Cui Y, Li X, Yao M. 2021. Microeco: an R package for data mining in microbial community ecology. FEMS Microbiol Ecol 97:fiaa255. doi:10.1093/femsec/fiaa255 33332530

[34] Segata N, Izard J, Waldron L, Gevers D, Miropolsky L, Garrett WS, Huttenhower C. 2011. Metagenomic biomarker discovery and explanation. Genome Biol 12:R60. doi:10.1186/gb-2011-12-6-r60 21702898 PMC3218848

[35] Carratalà A, Chappelier C, Selmoni O, Guillaume AS, Chmiel HE, Pasche N, Weil C, Kohn T, Joost S. 2023. Vertical distribution and seasonal dynamics of planktonic cyanobacteria communities in a water column of deep mesotrophic Lake Geneva. Front Microbiol 14:1295193. doi:10.3389/fmicb.2023.1295193 38169808 PMC10758419

[36] Wu J-W, Chen X-L. 2011. Extracellular metalloproteases from bacteria. Appl Microbiol Biotechnol 92:253–262. doi:10.1007/s00253-011-3532-8 21845384

[37] Rowell CER, Dobrovolny HM. 2020. Energy requirements for loss of viral infectivity. Food Environ Virol 12:281–294. doi:10.1007/s12560-020-09439-9 32757142 PMC7405386

[38] Lindsay RJ, Jepson A, Butt L, Holder PJ, Smug BJ, Gudelj I. 2021. Would that it were so simple: interactions between multiple traits undermine classical single-trait-based predictions of microbial community function and evolution. Ecol Lett 24:2775–2795. doi:10.1111/ele.13861 34453399

[39] Johnson DR, Helbling DE, Lee TK, Park J, Fenner K, Kohler H-P, Ackermann M. 2015. Association of biodiversity with the rates of micropollutant biotransformations among full-scale wastewater treatment plant communities. Appl Environ Microbiol 81:666–675. doi:10.1128/AEM.03286-14 25398862 PMC4277575

[40] Stadler LB, Delgado Vela J, Jain S, Dick GJ, Love NG. 2018. Elucidating the impact of microbial community biodiversity on pharmaceutical biotransformation during wastewater treatment. Microb Biotechnol 11:995–1007. doi:10.1111/1751-7915.12870 29076630 PMC6196385

[41] Wilhelm SW, Suttle CA. 1999. Viruses and nutrient cycles in the sea: viruses play critical roles in the structure and function of aquatic food webs. Bioscience 49:781–788. doi:10.2307/1313569

[42] Delgado‐Baquerizo M, Giaramida L, Reich PB, Khachane AN, Hamonts K, Edwards C, Lawton LA, Singh BK. 2016. Lack of functional redundancy in the relationship between microbial diversity and ecosystem functioning. J Ecol 104:936–946. doi:10.1111/1365-2745.12585

[43] Mao Z, Zhao Z, Da J, Tao Y, Li H, Zhao B, Xing P, Wu Q. 2023. The selection of copiotrophs may complicate biodiversity-ecosystem functioning relationships in microbial dilution-to-extinction experiments. Environ Microbiome 18:19. doi:10.1186/s40793-023-00478-w 36932455 PMC10024408

[44] Sangkhobol V, Skerman VBD. 1981. Chitinophaga, a new genus of chitinolytic myxobacteria. Int J Syst Bacteriol 31:285–293. doi:10.1099/00207713-31-3-285

[45] Rosenberg E. 2014. The family Chitinophagaceae, p 493–495. In Rosenberg E, DeLong EF, Lory S, Stackebrandt E, Thompson F (ed), The prokaryotes: other major lineages of bacteria and the archaea. Springer, Berlin, Heidelberg.

[46] Kishi LT, Lopes EM, Fernandes CC, Fernandes GC, Sacco LP, Carareto Alves LM, Lemos EGM. 2017. Draft genome sequence of a chitinophaga strain isolated from a lignocellulose biomass-degrading consortium. Genome Announc 5:01056–16. doi:10.1128/genomeA.01056-16 PMC5255924 28104646

[47] Fernandes GC, Sierra EGM, Brear P, Pereira MR, Lemos EGM. 2021. From data mining of Chitinophaga sp. genome to enzyme discovery of a hyperthermophilic metallocarboxypeptidase. Microorganisms 9:393. doi:10.3390/microorganisms9020393 33673011 PMC7918520

[48] Laily IN, Takeuchi M, Mizutani T, Ogawa J. 2023. An ACE2, SARS-CoV-2 spike protein binding protein, -like enzyme isolated from food-related microorganisms. Biosci Biotechnol Biochem 87:638–645. doi:10.1093/bbb/zbad037 36997336

[49] Mouillot D, Bellwood DR, Baraloto C, Chave J, Galzin R, Harmelin-Vivien M, Kulbicki M, Lavergne S, Lavorel S, Mouquet N, Paine CET, Renaud J, Thuiller W. 2013. Rare species support vulnerable functions in high-diversity ecosystems. PLoS Biol 11:e1001569. doi:10.1371/journal.pbio.1001569 23723735 PMC3665844

[50] Leitão RP, Zuanon J, Villéger S, Williams SE, Baraloto C, Fortunel C, Mendonça FP, Mouillot D. 2016. Rare species contribute disproportionately to the functional structure of species assemblages. Proc R Soc B 283:20160084. doi:10.1098/rspb.2016.0084 PMC4843652 27053754

