# Supplementary material for: Diversity of lake bacteria promotes human echovirus inactivation
Source: Appl Environ Microbiol. 2025 Jan 17;91(2):e02366-24. doi: 10.1128/aem.02366-24 (PMC11837565; doi:10.1128/aem.02366-24)
Supplement: Supplemental material — Figures S1 to S9; Table S1. [file aem.02366-24-s0001.pdf]

Supplementary Information

to

Diversity of lake bacteria promotes human echovirus inactivation

Andrii Romanenko, Hannes Peter, Josephine Meibom, Mark Borchardt, Tamar Kohn

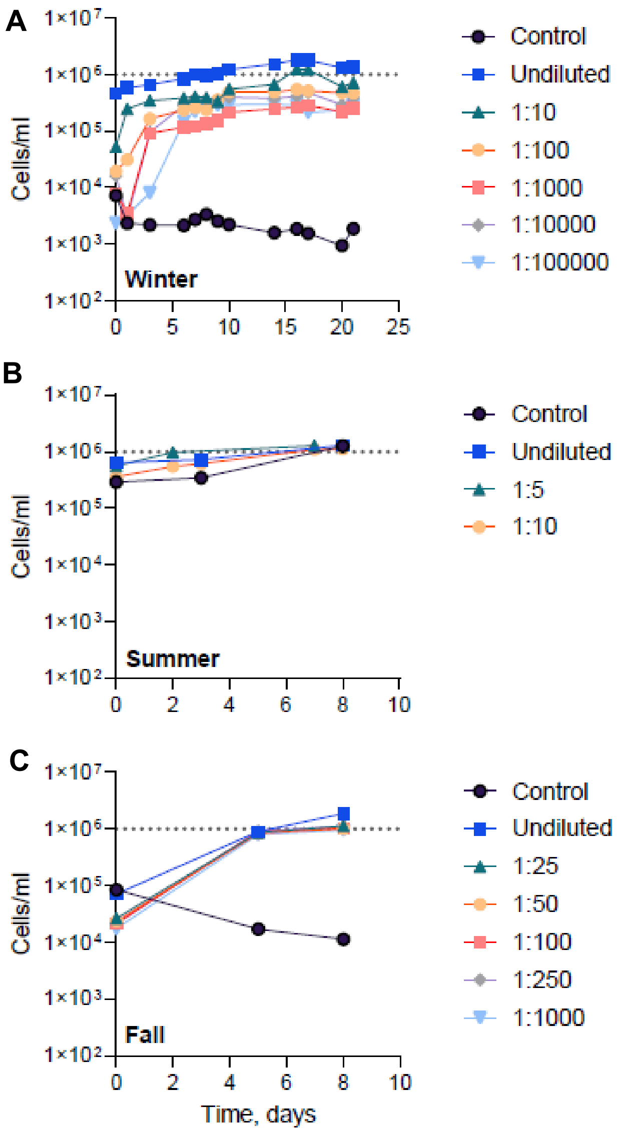

Supplementary Figure S1 Bacterial growth in dilutions in (A) winter, (B) summer, and (C) fall samples.

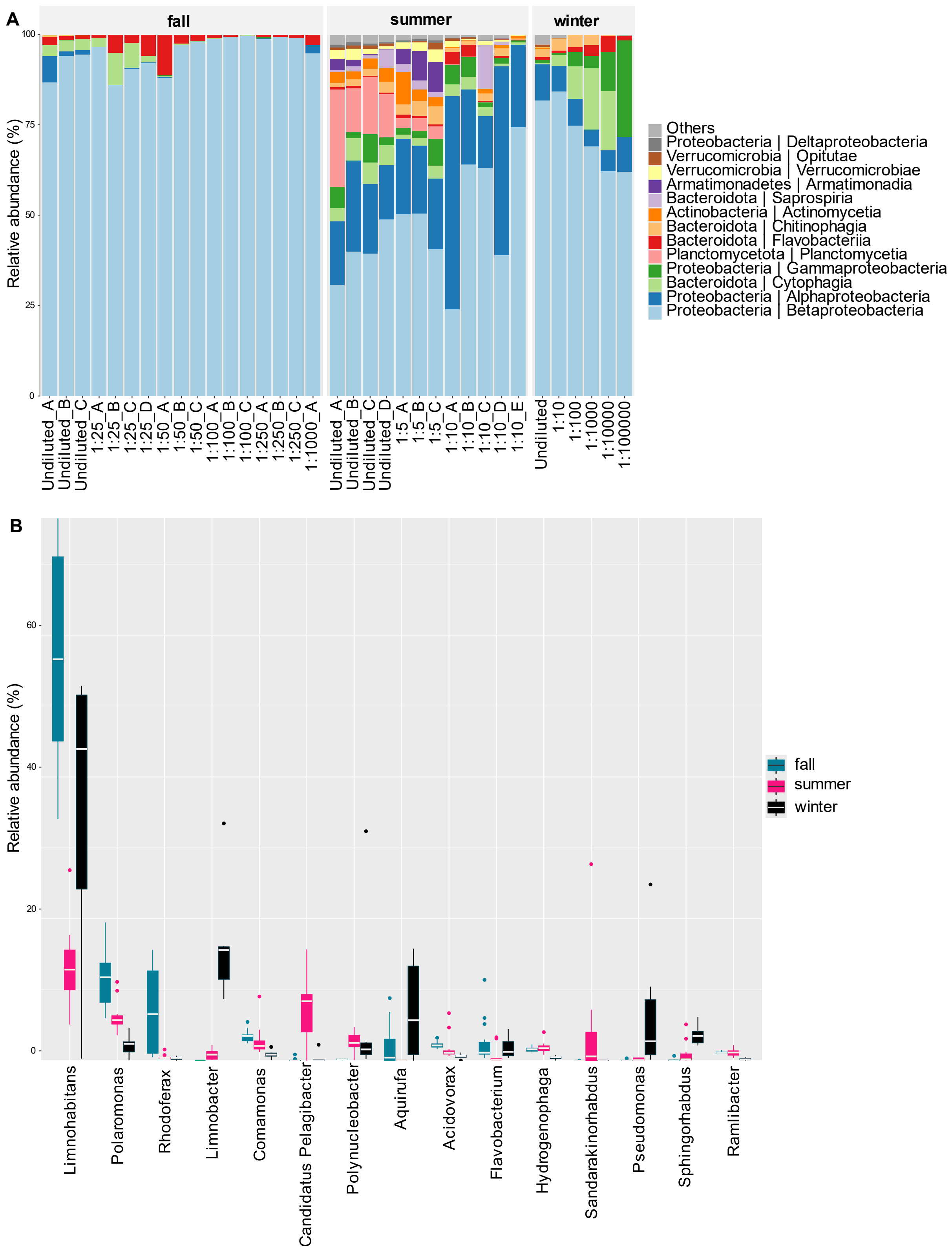

Supplementary Figure S2 Overview of taxonomic composition of samples along the dilution gradient and the different seasons (**A**). Taxonomy is resolved for the 13 most abundant classes. Boxplot showing average relative abundance of the most abundant genera (n=15) detected during different seasons (**B**).

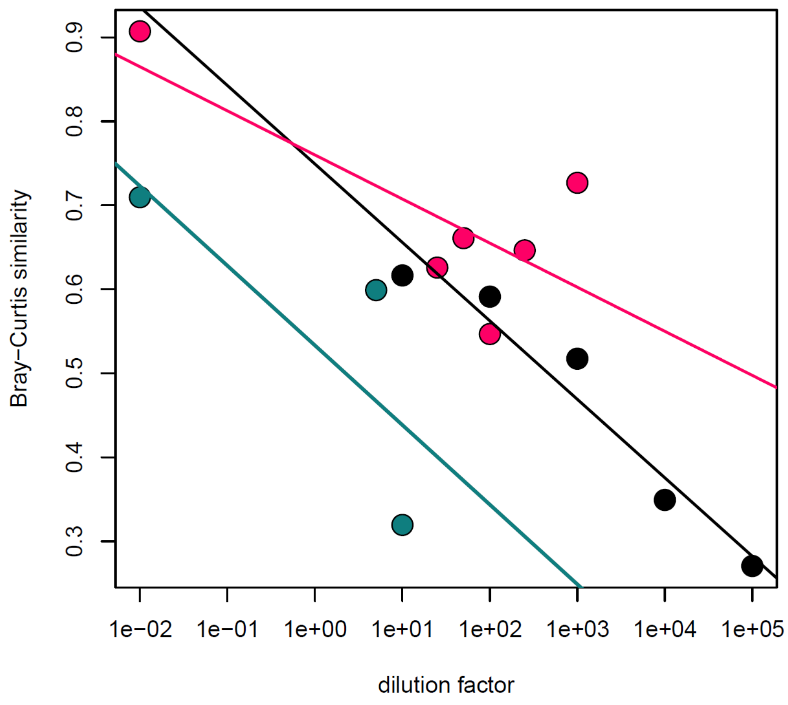

Supplementary Figure S3 Bray-Curtis similarity between diluted and undiluted samples in the three different seasons. Solid lines represent linear model fits, colors depict the three seasons (same as in SI Fig. S2). Note that increasing dilution lead to increasingly dissimilar bacterial communities across all seasons.

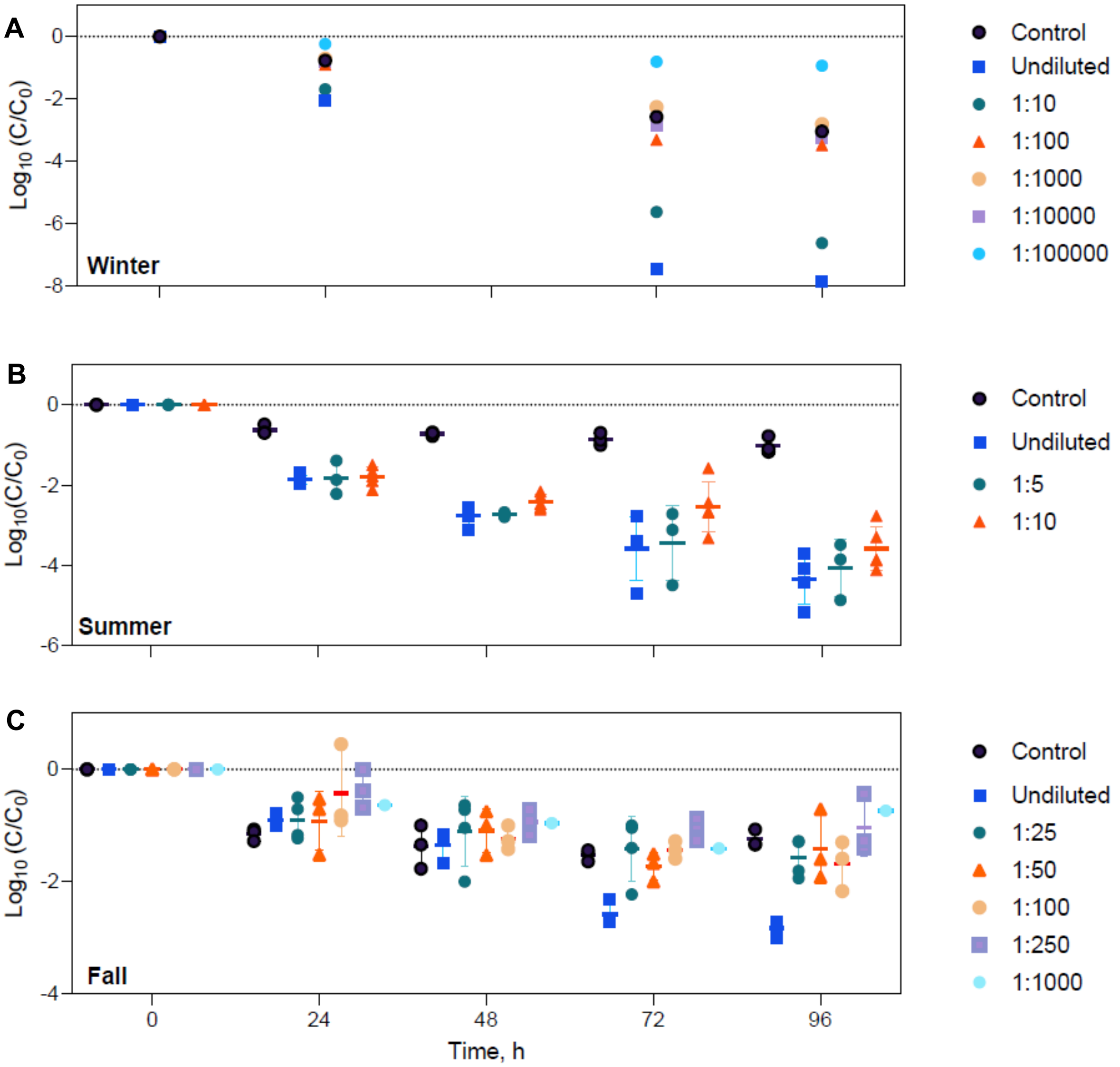

Supplementary Figure S4. Log-fold changes in inactivation of echovirus 11 in (A) winter, (B) summer, and (C) fall samples.

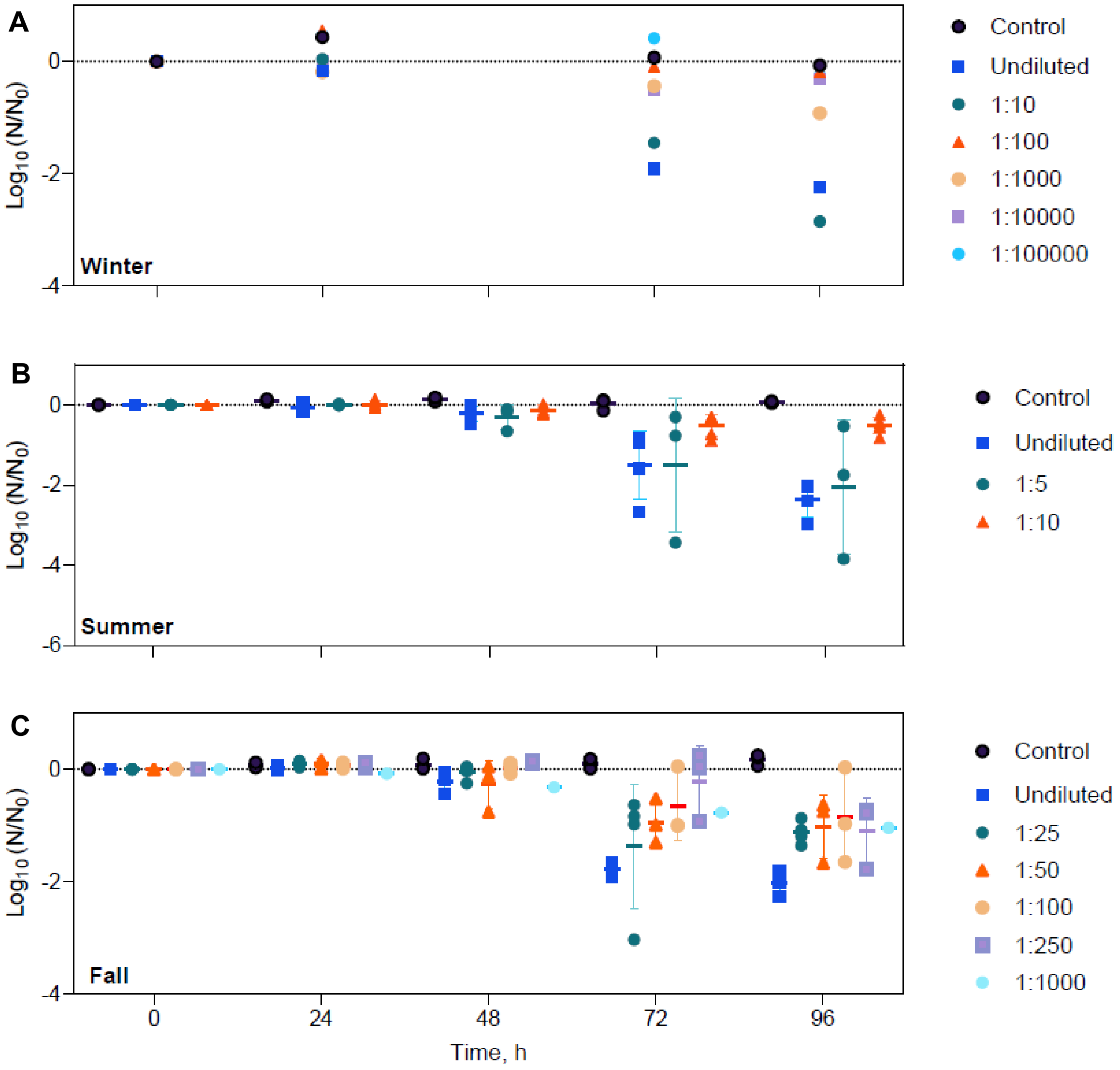

Supplementary Figure S5. Log-fold changes in genome decay of echovirus 11 in (A) winter, (B) summer, and (C) fall samples.

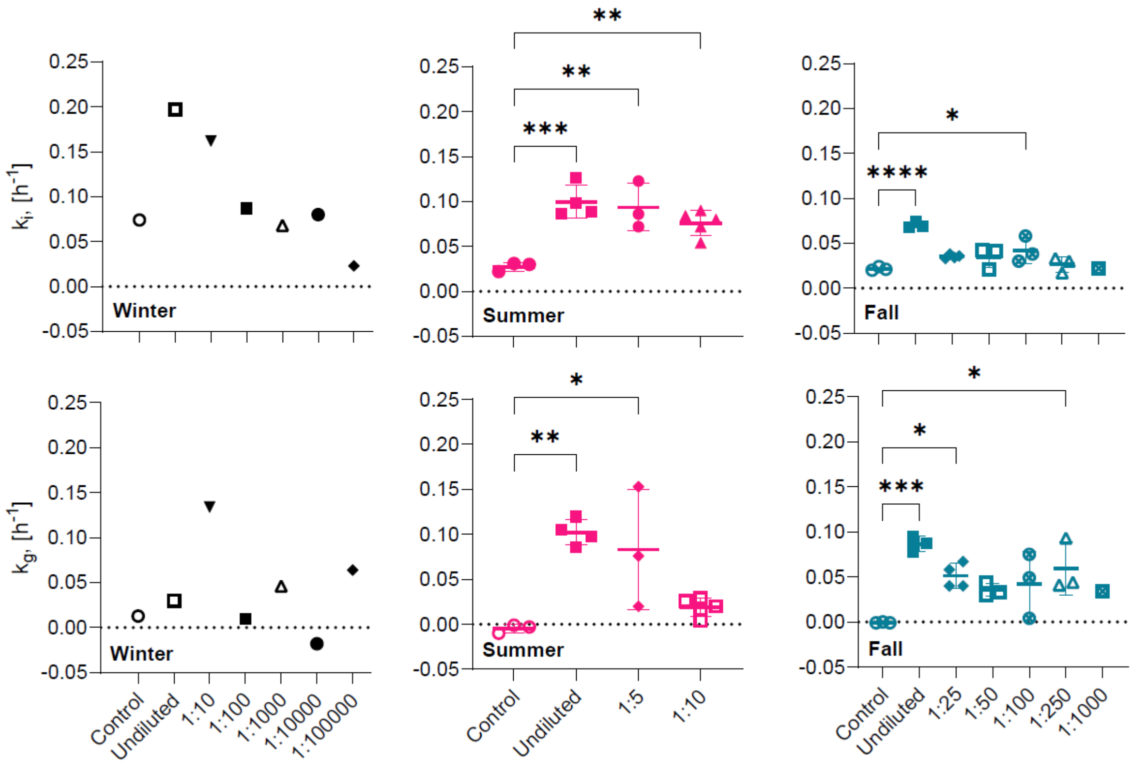

Supplementary Figure S6. Inactivation (k_i) (top) and genome (k_g) decay rates constants (bottom) as a function of dilution for winter, summer, and fall samples. Significance testing was done using ordinary one-way ANOVA with Dunnet's test to correct for multiple comparisons. * indicates P <0.05, ** P <0.01, *** P <0.001. Colors depict different seasons (same as in SI Fig. 2).

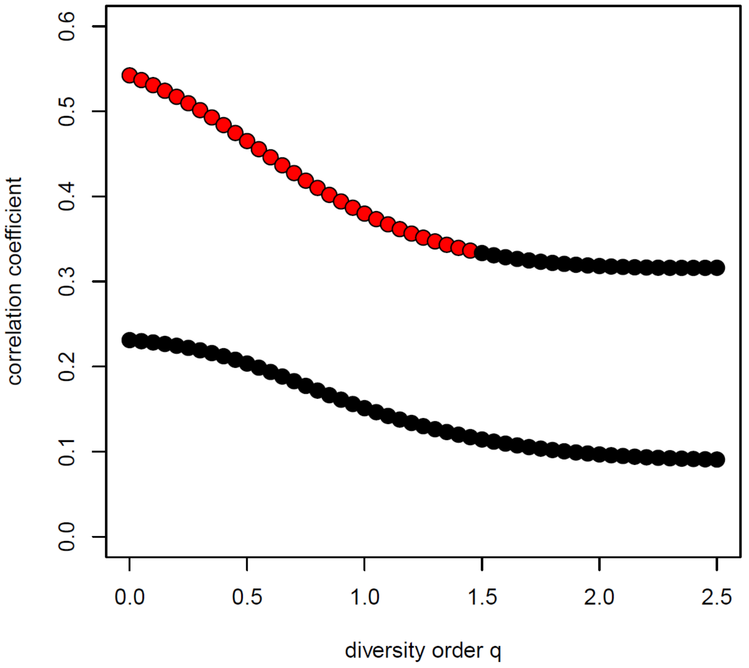

Supplementary Figure S7. Shown are correlation coefficients between inactivation rate constants (upper line) and genome decay rate constants (lower line) and diversity estimates of order q. Significant correlations are shown in red, non-significant correlations (P >0.05) are shown in black. Hill number (effective number of species) estimates of increasing order q emphasize abundant species over rare species. For instance, at q=0 all species, irrespective if rare or abundant, are considered and shown are the correlation coefficients between species richness and inactivation and genome decay rate constants, respectively. At q=1 (which equals Shannon diversity estimate), communities harboring more abundant species are considered more diverse, whereas rare species do not contribute as much to diversity. Overall this analysis shows that strong positive diversity-inactivation relationships are only detected when using diversity estimates of low order q (q<1.5), suggesting that rare species play an important role in the viral inactivation.

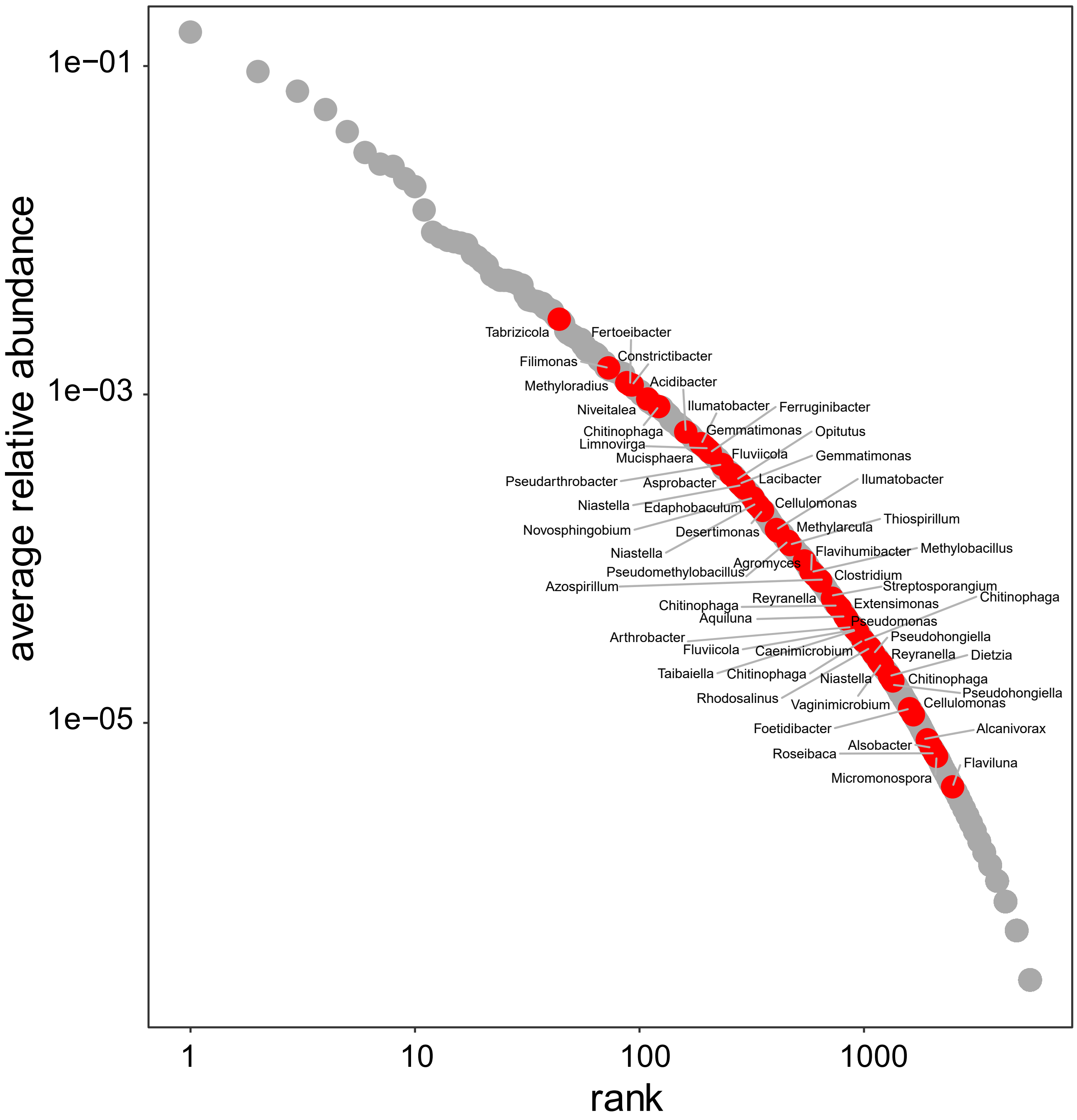

Supplementary Figure S8 A fully annotated version of the rank-abundance distribution. Red symbols depict the rank and average abundance of 61 bacterial species identified as biomarkers for high echovirus 11 inactivation using LEfSe.

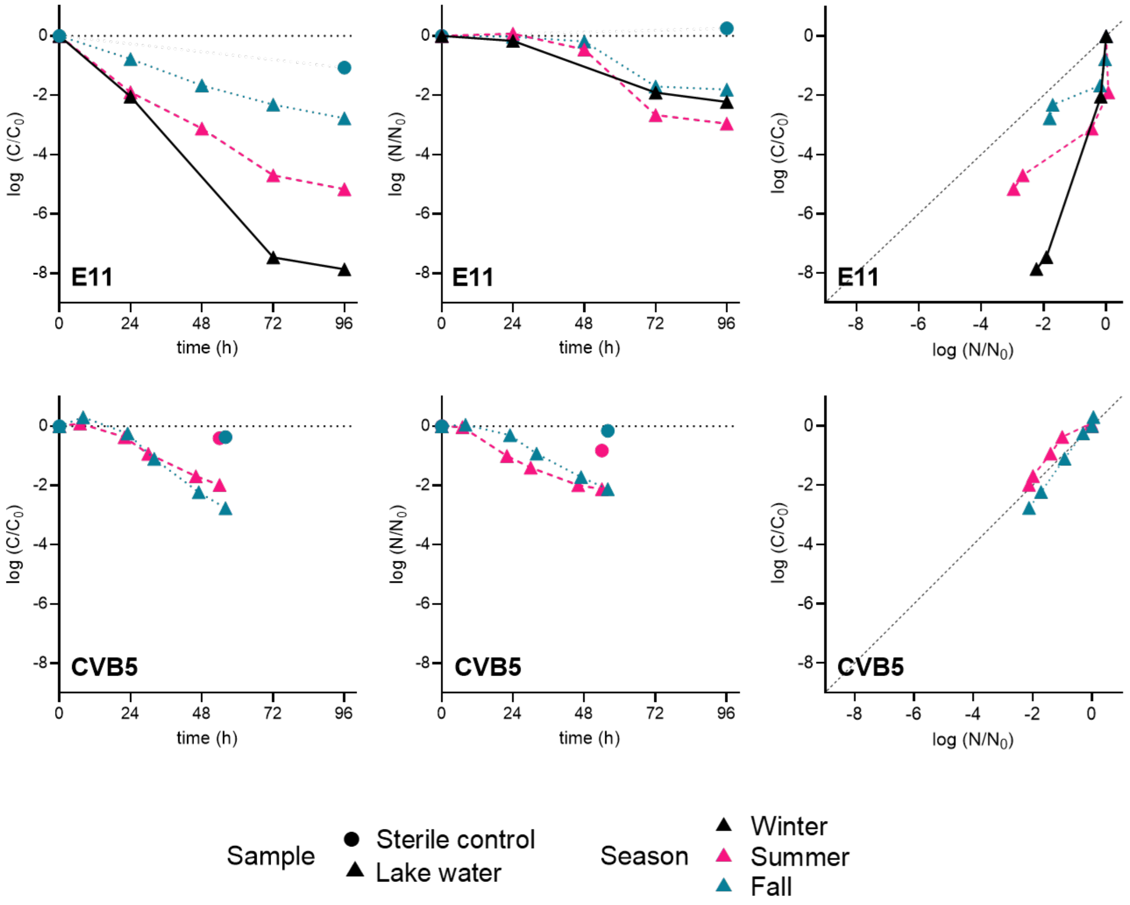

Supplementary Figure S9 Comparison of inactivation and genome decay kinetics for echovirus 11 (E11) and coxsackievirus B5 (CVB5). Left panels: inactivation in microbially active, undiluted lake water samples and sterile controls; middle panels: genome decay in microbially active, undiluted lake water samples and sterile controls; right panels: inactivation vs. genome decay. The dotted line in the right panels indicates the unity line. For echovirus 11, inactivation proceeds faster than genome decay. For coxsackievirus B5, data points fall on the unity line, indicating equivalent kinetics for inactivation and genome decay.

Echovirus 11 data correspond to those shown in Figure 2 of the main text. Coxsackievirus B5 experiments were conducted in water samples obtained on July 10, 2023 (summer) and September 11, 2023 (fall). Coxsackievirus B5 was isolated from Lausanne wastewater and corresponded to isolate CVB5-L060815 described elsewhere (1) and was propagated using the same protocol as for echovirus 11 described in the main text. Aliquots of 50 mL (sterile) lake water were amended with coxsackievirus B5 to a concentration of 10^7 MPN/ml. All spiked samples were incubated at room temperature on a shaking table. Two 0.5 mL aliquots were taken twice daily throughout the virus decay experiment and stored at -20°C until enumeration. Aliquots of the sterile lake water samples were only taken at the start and end of the decay experiment. The residual virus concentration in each sample was determined by both MPN assay and RT-dPCR using the same assays as described for echovirus 11 in the main text. Data points represent the average of triplicate experiments.

Supplementary Table S1. List of 61 bacterial biomarkers and higher-level taxonomies associated with high echovirus 11 inactivation rates.

ID	Mean relative abundance (%)	rank	Species	Phylum	Class
ID_3624	0.29	44	Tabrizicola oligotrophica	Proteobacteria	Alphaproteobacteria
ID_1092	0.15	73	Filimonas zeae	Bacteroidota	Chitinophagia
ID_5001	0.12	88	Methyloradius palustris	Proteobacteria	Betaproteobacteria
ID_3453	0.11	91	Fertoeibacter niger	Proteobacteria	Alphaproteobacteria
ID_3894	0.11	93	Constrictibacter antarcticus	Proteobacteria	Alphaproteobacteria
ID_1152	0.09	109	Niveitalea solisilvae	Bacteroidota	Chitinophagia
ID_1058	0.08	122	Chitinophaga eiseniae	Bacteroidota	Chitinophagia
ID_5653	0.06	161	Acidibacter ferrireducens	Proteobacteria	Gammaproteobacteria
ID_2755	0.05	188	Mucisphaera calidilacus	Planctomycetota	Phycisphaerae
ID_40	0.05	189	Ilumatobacter fluminis	Actinobacteria	Acidimicrobiia
ID_1133	0.05	195	Limnovirga soli	Bacteroidota	Chitinophagia
ID_2742	0.05	203	Gemmatimonas phototrophica	Gemmatimonadetes	Gemmatimonadetes
ID_1087	0.04	207	Ferruginibacter yonginensis	Bacteroidota	Chitinophagia
ID_453	0.04	234	Pseudarthrobacter scleromae	Actinobacteria	Actinomycetia
ID_1447	0.03	256	Fluviicola taffensis	Bacteroidota	Flavobacteriia
ID_3378	0.03	259	Asprobacter aquaticus	Proteobacteria	Alphaproteobacteria
ID_6449	0.03	271	Opitutus terrae	Verrucomicrobia	Opitutae
ID_2741	0.03	280	Gemmatimonas aurantiaca	Gemmatimonadetes	Gemmatimonadetes
ID_1130	0.03	284	Lacibacter daechungensis	Bacteroidota	Chitinophagia
ID_1149	0.03	285	Niastella populi	Bacteroidota	Chitinophagia
ID_1082	0.03	293	Edaphobaculum flavum	Bacteroidota	Chitinophagia
ID_4054	0.02	321	Novosphingobium lentum	Proteobacteria	Alphaproteobacteria
ID_1148	0.02	332	Niastella koreensis	Bacteroidota	Chitinophagia
ID_221	0.02	339	Cellulomonas endophytica	Actinobacteria	Actinomycetia
ID_38	0.02	354	Desertimonas flava	Actinobacteria	Acidimicrobiia
ID_41	0.02	408	Ilumatobacter nonamiensis	Actinobacteria	Acidimicrobiia
ID_3490	0.01	414	Methylarcula terricola	Proteobacteria	Alphaproteobacteria
ID_5009	0.01	460	Pseudomethylobacillus aquaticus	Proteobacteria	Betaproteobacteria
ID_5513	0.01	469	Thiospirillum jenense	Proteobacteria	Gammaproteobacteria
ID_292	0.01	544	Agromyces agglutinans	Actinobacteria	Actinomycetia
ID_1101	0.01	583	Flavihumibacter soli	Bacteroidota	Chitinophagia
ID_4992	0.01	585	Methylobacillus pratensis	Proteobacteria	Betaproteobacteria
ID_2487	0.01	606	Clostridium manihotivorum	Firmicutes	Clostridia
ID_3807	0.01	637	Azospirillum canadense	Proteobacteria	Alphaproteobacteria
ID_3244	0.01	643	Reyranella massiliensis	Proteobacteria	Alphaproteobacteria
ID_910	0.01	725	Streptosporangium subfuscum	Actinobacteria	Actinomycetia
ID_1063	0.01	761	Chitinophaga oryziterrae	Bacteroidota	Chitinophagia
ID_4632	0.005	785	Extensimonas perlucida	Proteobacteria	Betaproteobacteria
ID_309	0.004	826	Candidatus Aquiluna rubra	Actinobacteria	Actinomycetia
ID_3135	0.004	843	Pseudomonas carboxydohydrogena	Proteobacteria	Alphaproteobacteria
ID_420	0.004	915	Arthrobacter roseus	Actinobacteria	Actinomycetia
ID_1445	0.004	920	Fluviicola hefeinensis	Bacteroidota	Flavobacteriia
ID_1183	0.004	923	Taibaiella yonginensis	Bacteroidota	Chitinophagia
ID_4351	0.004	939	Caenimicrobium hargitense	Proteobacteria	Betaproteobacteria
ID_1055	0.003	992	Chitinophaga cymbidii	Bacteroidota	Chitinophagia
ID_1065	0.003	998	Chitinophaga pinensis	Bacteroidota	Chitinophagia
ID_3596	0.003	1066	Rhodosalinus sediminis	Proteobacteria	Alphaproteobacteria
ID_5662	0.003	1106	Pseudohongiella acticola	Proteobacteria	Gammaproteobacteria
ID_1151	0.002	1165	Niastella vici	Bacteroidota	Chitinophagia
ID_633	0.002	1198	Vaginimicrobium propionicum	Actinobacteria	Actinomycetia
ID_3246	0.002	1213	Reyranella terrae	Proteobacteria	Alphaproteobacteria
ID_117	0.002	1302	Dietzia psychralcaliphila	Actinobacteria	Actinomycetia
ID_1057	0.002	1339	Chitinophaga dinghuensis	Bacteroidota	Chitinophagia
ID_5663	0.002	1347	Pseudohongiella nitratireducens	Proteobacteria	Gammaproteobacteria
ID_1119	0.001	1600	Foetidibacter luteolus	Bacteroidota	Chitinophagia
ID_226	0.001	1664	Cellulomonas phragmiteti	Actinobacteria	Actinomycetia
ID_5855	0.001	1919	Alcanivorax profundi	Proteobacteria	Gammaproteobacteria
ID_2921	0.001	1999	Alsobacter metallidurans	Proteobacteria	Alphaproteobacteria
ID_3607	0.001	2069	Roseibaca ekhonensis	Proteobacteria	Alphaproteobacteria
ID_529	0.001	2110	Micromonospora globbae	Actinobacteria	Actinomycetia
ID_313	0.0004	2487	Candidatus Flaviluna lacus	Actinobacteria	Actinomycetia

References

(1) Meister S, Verbyla ME, Klinger M, Kohn T. 2018. Variability in Disinfection Resistance between Currently Circulating Enterovirus B Serotypes and Strains. Environ Sci Technol 52:3696–3705.
